# Longitudinal changes in the vaginal microbiome associate with spontaneous preterm birth: a focus on stability and specific taxa in a Chinese cohort

**DOI:** 10.1128/spectrum.03705-25

**Published:** 2026-07-17

**Authors:** Yi Yu, Jingwen Zhou, Qian Zhou, Xu Li, Lanying Zhang, Sabrina Li, Baoli Zhu, Jinsong Gao, Juntao Liu

**Affiliations:** 1 Department of Obstetrics and Gynecology, Peking Union Medical College Hospital, Chinese Academy of Medical Sciences, Peking Union Medical College, National Clinical Research Center for Obstetric and Gynecologic Diseases 570034, Beijing, China; 2 COYOTE Medical Laboratory, Beijing, China; 3 CAS Key Laboratory of Pathogenic Microbiology and Immunology, Institute of Microbiology, Chinese Academy of Science 85387, Beijing, China; Third Affiliated Hospital of Sun Yat-sen University, Guangzhou, China; Northwest A&F University, Yangling, China

**Keywords:** vaginal microbiome, real-time polymerase chain reaction, preterm birth, *Lactobacillus *spp., *Ureaplasma *spp., microbial stability

## Abstract

Spontaneous preterm birth (sPTB) often results from ascending intra-amniotic infections originating from the vaginal microbiota. This study aimed to characterize vaginal microbial features and identify specific taxa associated with sPTB in a Chinese population. In this prospective cohort study, pregnant women were recruited from Peking Union Medical College Hospital. Vaginal swabs were collected longitudinally at 11–16, 22–28, and 34–37 weeks of gestation. DNA was analyzed using targeted real-time PCR (30 pathogens) and 16S rRNA gene sequencing (V3–V4). Community State Types (CSTs) were defined by enterotype-like clustering. Differential abundance was assessed using MaAsLin3 with Benjamini-Hochberg correction, adjusting for maternal age, BMI, and obstetric history. Among 273 women (26 sPTB, 247 term), no CST was significantly associated with sPTB after multivariable adjustment and false discovery rate (FDR) correction (all *q* > 0.05). Alpha and beta diversity also showed no significant between-group differences (all *q* > 0.05). However, longitudinal CST stability was significantly lower in the sPTB group (*P* < 0.05). Nominally significant associations were observed for *Ureaplasma urealyticum* (first trimester), *Mycoplasma hominis*, and *Bacteroides fragilis* (second trimester), but none survived FDR correction (all *q* > 0.05). In this Chinese cohort, sPTB was not associated with static CST profiles but with reduced microbial stability over time. Although no single taxon remained significant after correction, several pathogens showed nominal associations, warranting further investigation in larger studies.

**IMPORTANCE:**

Preterm birth (PTB) is a global maternal and infant health issue affecting approximately 11% of newborns worldwide. In China, the prevalence of PTB was 6.1%. Abnormal vaginal microbiota has been demonstrated to be a risk factor for PTB. Our study is one of the largest studies performed to date to investigate the associations between vaginal microbiome and spontaneous PTB (sPTB) in the Chinese cohort. We found that vaginal microbiome dynamics changes in Community State Types (CSTs) were significantly associated with sPTB. Furthermore, we also found that the microbial risk for sPTB appeared to be the enrichment of specific taxa, suggesting that vaginal dynamics and fine-scale features are important factors to consider in future studies.

## INTRODUCTION

Preterm birth (PTB) remains a critical global health challenge. In China, its prevalence is 6.1% (1), posing significant risks for neonatal complications and mortality (2). A leading cause of spontaneous PTB (sPTB) is ascending intra-amniotic infection from the vagina (3–5), highlighting the crucial role of the vaginal microbiome in pregnancy health (6, 7).

A healthy vaginal environment is typically dominated by *Lactobacillus* species, which maintain a protective, low-pH environment. Conversely, bacterial vaginosis (BV), characterized by a decline in lactobacilli and an increase in diverse anaerobic bacteria, is a recognized risk factor for PTB (8, 9). The Community State Type (CST) classification system, particularly CST-IV (a *Lactobacillus*-poor, high-diversity state), has been linked to sPTB in some studies (10–12). However, this association is inconsistent across populations (13, 14), suggesting substantial heterogeneity influenced by race, geography, and methodology.

This inconsistency prompts a shift in research focus from broad community types to specific microbial markers. Predictive models based on precise bacterial taxa show promise but also exhibit variations (15, 16). In recent years, several studies have proved the relationship between the vaginal microbiome and PTB in Chinese populations (17–20). However, the findings have been notably inconsistent, particularly regarding alpha diversity and specific taxa associated with sPTB. More importantly, all existing studies in Chinese populations to date have employed cross-sectional or case-control designs with single-time-point sampling. While these studies establish that associations exist, they cannot capture the dynamic nature of the vaginal microbiome during pregnancy or determine whether temporal instability, rather than a static community state, contributes to sPTB risk.

To address this gap, we conducted a prospective longitudinal cohort study in a Chinese population. We employed a dual-method approach (16S rRNA sequencing and targeted qPCR) across three trimesters to characterize the vaginal microbiome’s dynamic relationship with sPTB. Moving beyond the traditional CST framework, which often yields inconsistent associations, we focus on identifying some more specific microbial species and quantities, as well as the temporal stability of the vaginal microbiome across pregnancy associated with sPTB.

## MATERIALS AND METHODS

### Study design and patient enrollment

This prospective cohort study was conducted at the Peking Union Medical College Hospital (PUMCH) between December 2019 and September 2021. Pregnant women receiving routine prenatal care at our hospital were enrolled at a gestational age of 8^+0^ to 13^+6^ weeks. Gestational age was determined based on the last menstrual period and confirmed by an ultrasound examination before 24 weeks (preferably by crown-rump length in the first trimester). The exclusion criteria included the administration of antibiotics within 1 month prior to enrollment or pregnancy termination. Medical and obstetric history, obstetric events during pregnancy, and pregnancy outcomes were collected from medical records.

PTB was defined as delivery from 20^+0^ to 36^+6^ weeks of gestation. Our analysis focused specifically on sPTB, defined as birth subsequent to spontaneous labor or preterm premature rupture of the membranes (PPROM). All iatrogenic (medically indicated) PTBs were excluded.

### Vaginal sample collection and DNA extraction

Maternal vaginal swabs were collected longitudinally during routine prenatal visits at the following gestational windows: 11–16 weeks, 22–28 weeks, and 34–37 weeks. The sample was collected from the posterior fornix using a sterile swab (Starplex Scientific, Ontario, Canada) under a vaginal speculum performed by a trained obstetrician. The swab was placed in a sterile collection tube with 1 mL of modified Liquid Amies solution and stored at −20°C prior to nucleic acid extraction. Total genomic DNA was extracted using the QIAamp DNA Microbiome Kit (Qiagen, Hilden, Germany) according to the manufacturer’s protocol for microbial analysis. DNA concentration was determined using a Qubit 3.0 Fluorometer (Q32866; Life Technologies, Carlsbad, CA).

### Vaginal microbes detection by qPCR and 16S rRNA gene sequencing

Vaginal microbial analysis was conducted using two complementary approaches: a targeted qPCR-based assay and broad-range 16S rRNA gene sequencing. The targeted detection was performed using the FlashDetect MAX vaginal microbes detection kit (30Panel; Coyote, Beijing, China), which enables the simultaneous identification and quantification of 30 vaginal microorganisms via real-time PCR. The panel covers pathogens associated with bacterial vaginosis, aerobic vaginitis, vulvovaginal candidiasis, trichomoniasis, chlamydia, mycoplasma, and other sexually transmitted infections.

For qPCR quantification, a plasmid containing the specific microbial targets and 16S PCR amplicon from *Escherichia coli* was serially diluted from 10^7^ to 10 copies to generate a standard curve and determine qPCR efficiency. The concentrations of microorganisms and 16S were derived from the measured Ct values, enabling subsequent calculation of their relative percentages in the samples.

For broad-range microbial profiling, next-generation sequencing of the bacterial 16S rRNA gene was performed. The hypervariable V3–V4 regions were amplified using primer-F (5′-CCTAYGGGRBGCASCAG-3′) and primer-R (5′-GGACTACNNGGGTATCTAAT-3′). DNA templates were amplified using 1× KAPA HiFi Master Mix (Cat. No. 16SAFP02; Coyote Biosciences, Beijing, China), 0.01 nmol of each primer, and 9 μL genomic DNA. Amplification products were purified with VAHTS clean beads (Na44-02; Coyote) and quantified with a Qubit dsDNA HS Assay Kit (Q32851; Life Technologies). Amplicon libraries were prepared, purified, and paired-end sequenced (2 × 250 bp) on an Illumina HiSeq 2500 platform (Illumina Inc., San Diego, CA).

### Bioinformatics and statistical analysis

Raw 16S rRNA gene sequences were processed using QIIME2 (version 2022.8) (21). High-quality sequences were obtained after quality filtering, merging of paired-end reads, and removal of chimeras. Taxonomic information was assigned to amplicon sequence variants by alignment against the SILVA 138 reference database. The *Lactobacillus* species was cross-validated via BLAST search against the NCBI 16S rRNA database (https://ftp.ncbi.nlm.nih.gov/blast/db/16S_ribosomal_RNA.tar.gz).

CST classification was determined through enterotype-like clustering tailored to our data set. First, species-level taxonomic profiles were generated. Subsequently, hierarchical clustering based on Bray-Curtis distance matrices with Ward.D2 linkage was applied to group compositionally similar samples into distinct CSTs (22). The CST definitions are tailored to our study population rather than being forced into pre-defined categories from other cohorts.

Alpha diversity was assessed using the Chao1, Shannon, and Simpson indices. Differences in alpha diversity between groups across trimesters were evaluated with the Kruskal-Wallis test, with Benjamini-Hochberg (BH) false discovery rate (FDR) correction applied to account for multiple comparisons. Beta diversity was evaluated using Bray-Curtis dissimilarity, visualized via principal coordinate analysis (PCoA) and non-metric multidimensional scaling (NMDS) plots. Analysis of similarity (ANOSIM) was used to determine statistical differences in beta diversity, with FDR correction applied across comparisons. To identify taxa driving the separation observed in PCoA plots, we calculated species loadings on the principal coordinates by correlating the relative abundance of each taxon with sample coordinates. These loadings were visualized as vectors in a compositional biplot.

Differential abundance analysis was performed using MaAsLin3 with BH FDR correction. The model included maternal age, BMI, and history of adverse obstetric outcomes (sPTB, late miscarriage, cervical insufficiency) as covariates to adjust for potential confounders. Analyses were performed separately for abundance data (continuous) and prevalence data (binary). A corrected *q*-value < 0.1 was considered statistically significant, with *q* < 0.05 considered strongly significant. CST association with pregnancy outcome was assessed using multivariable logistic regression models implemented in MaAsLin3, controlling for the same covariates. FDR correction was applied across all CST types within each trimester.

To illustrate longitudinal instability, stacked area graphs of community composition were generated for all samples and for representative sPTB and term controls. Sankey diagrams were created to visualize CST transitions across trimesters for all participants. All visualizations were generated using R (version 4.1.0) with ggplot2 and ggalluvial packages.

## RESULTS

### Characteristics of the study participants

The initial cohort enrolled 282 women. After excluding patients lost to follow-up (*n* = 3), those with pregnancy termination (*n* = 2), and iatrogenic PTBs (*n* = 4), 273 women remained for analysis, including 26 in the sPTB group and 247 in the term delivery group. The overall prevalence of sPTB was 9.5%. Among the sPTB cases, 26.9% (7/26) had PPROM, and 53.8% (14/26) were early PTB (<34 weeks). The demographic and clinical characteristics are summarized in Table 1. Significant differences were found between the two groups in maternal age, BMI, gestational weight gain, and history of adverse obstetric outcomes, such as sPTB, late miscarriage, and cervical insufficiency (*P* < 0.05). No significant differences were observed in parity, pregnancy-induced hypertension, gestational diabetes, or delivery mode.

**TABLE 1:** Demographic and birth characteristics of women in the study (data were given as mean ± SD or *n* [%])

Characteristics	Spontaneous preterm (*n* = 26)	Term (*n* = 247)	*P* value^*b*^
Maternal age, y	35.115 ± 4.033	33.093 ± 3.955	<0.001
Prenatal body mass index, kg/m^2^	22.095 ± 3.304	21.381 ± 3.056	<0.001
Weight gain during present pregnancy, kg	10.158 ± 4.018	12.696 ± 4.193	<0.001
Body mass index at delivery, kg/m^2^	25.878 ± 3.361	26.089 ± 3.462	<0.001
Parity			
Nulliparous	15 (57.6)	176 (71.3)	<0.001
Parous	11 (42.4)	71 (28.7)	
History of sPTB^*a*^	5 (45.5)	7 (9.9)	0.008
History of late spontaneous miscarriage	3 (11.5)	3 (1.2)	0.007
History of cervical insufficiency	6 (23.1)	5 (2.0)	<0.001
Assisted reproduction	7 (26.9)	25 (10.1)	0.027
Cervical cerclage during present pregnancy	6 (23.1)	5 (2.0)	<0.001
Premature rupture of membranes	7 (26.9)	36 (14.6)	0.016
Threatened late miscarriage	3 (11.5)	8 (3.2)	0.127
Threatened preterm birth	4 (15.3)	5 (2.0)	0.002
Pregnancy-induced hypertension	1 (5.3)	9 (3.6)	0.788
Gestational diabetes	8 (30.7)	64 (25.9)	0.223
Delivery mode			
Vaginal delivery	15 (57.6)	140 (56.7)	0.913
Cesarean section	11 (42.4)	107 (43.3)	
Gestational age at birth, wk^*c*^	31.159 ± 5.041	39.354 ± 0.999	<0.001
20 + 0 − 27 + 6	9 (34.6)	/^*d*^	
28 + 0 − 33 + 6	5 (19.2)	/	
34 + 0 − 36 + 6	12 (46.2)	/	
≥37	/	247	

^*a*^ Parous women only.

^*b*^ Significant at *P* < 0.05. *P* values are uncorrected for multiple testing and are presented for descriptive purposes to identify potential confounders for multivariable adjustment.

^*c*^ Analyzed by Student’s *t*-test as a numerical variable and by Chi-square test as a categorical variable.

^*d*^ /, not applicable.

### Vaginal microbiome dynamics and stability

**Fig 1:**
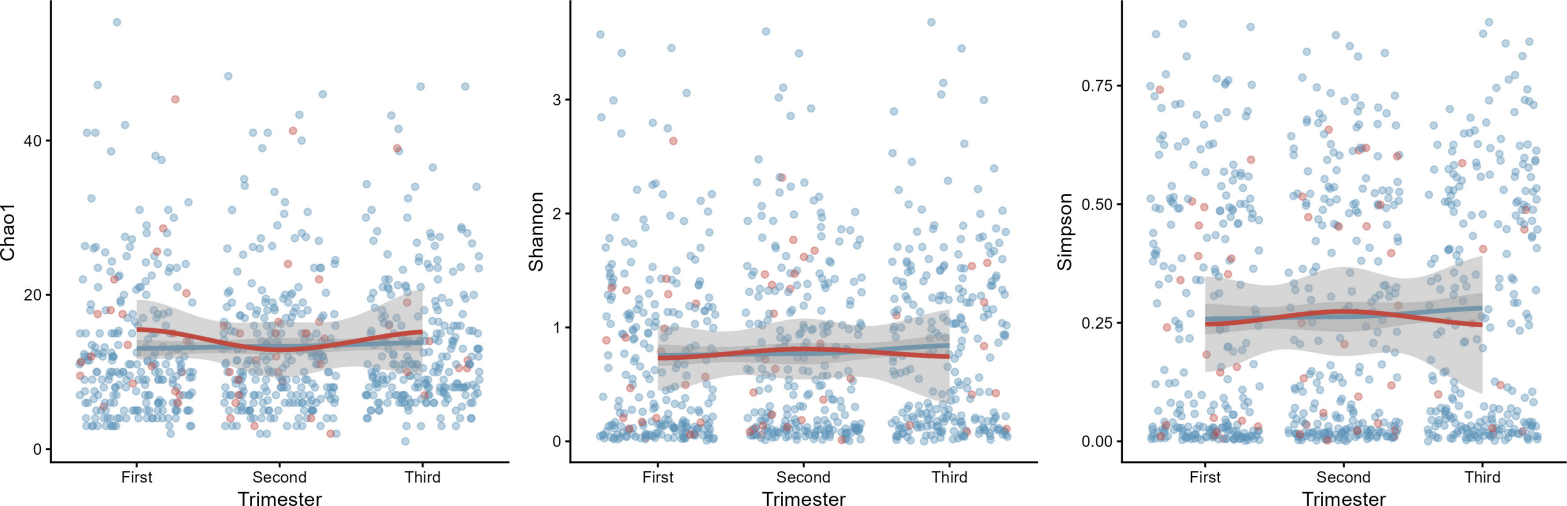
Alpha diversity across trimesters. Chao1, Shannon, and Simpson indices for term (blue, *n* = 235–247) and sPTB (red, *n* = 10–24) groups. No significant differences between groups at any trimester (Kruskal-Wallis with FDR correction, all *q* > 0.05).

Microbiome profiles generated by 16S rRNA taxonomic analysis using QIIME2 revealed the dynamic nature of the vaginal community during pregnancy. Figure 1 displays the alpha diversity (Chao1, Shannon, and Simpson diversity indices) across trimesters. There were no significant differences between term and sPTB groups in any alpha diversity metric at any trimester after FDR correction (all *q* > 0.05). Within-group comparisons, no significant longitudinal changes were observed in two groups for any metric (all *q* > 0.05).

**Fig 2:**
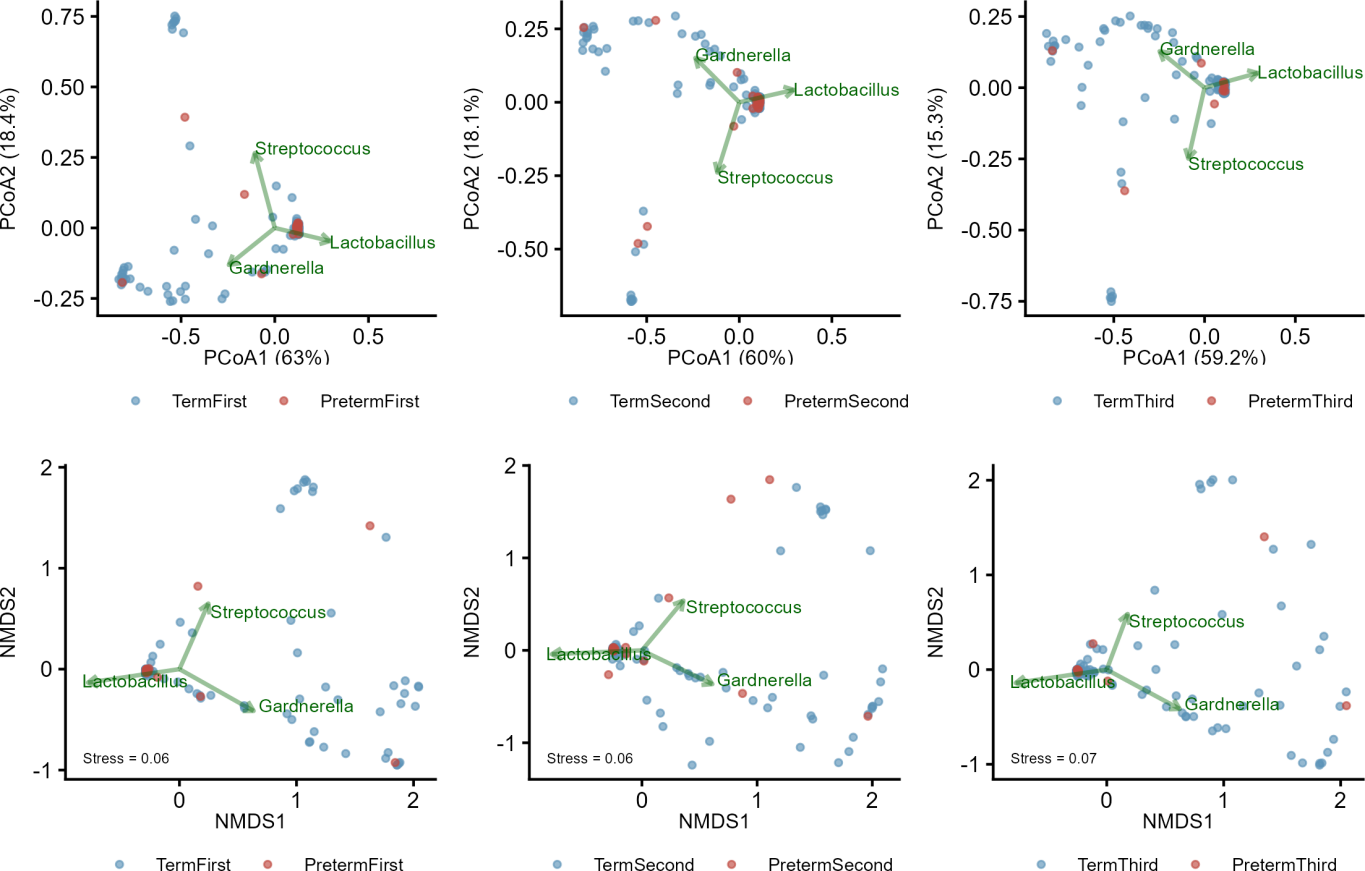
Beta diversity analysis (Bray-Curtis). PCoA and NMDS plots per trimester showing term (blue) and sPTB (red) samples. Sample sizes: first (term *n* = 235, sPTB *n* = 21), second (term *n* = 246, sPTB *n* = 24), third (term *n* = 243, sPTB *n* = 10). Variance explained in parentheses. ANOSIM R statistics with 95% CI: first (*P* = 0.387, *q* = 0.536), second (*P* = 0.117, *q* = 0.351), third (*P* = 0.059, *q* = 0.315) were not significant. Three clusters driven by (1) *Lactobacillus* dominance, (2) *Gardnerella* dominance, (3) *Streptococcus*/other. Distribution of term/sPTB across clusters did not differ (ANOSIM *P* > 0.05; Table 2).

Regarding the analysis of beta diversity using Bray-Curtis dissimilarity between the sPTB group compared to term controls, no significant differences were observed in the first (*P* = 0.387, *q* = 0.536), second (*P* = 0.117, *q* = 0.351), or the third trimester (*P* = 0.059, *q* = 0.315). PCoA and NMDS visualization (Fig. 2) showed substantial overlap between term and sPTB groups at all time points. Within-group comparisons revealed no significant differences in community composition across trimesters for either the term or sPTB group (all *q* > 0.05). However, the visual inspection of the PCoA and NMDS plots revealed a consistent separation of samples into three main clusters across all trimesters (Fig. 2). To identify the drivers of this separation, we examined species loadings on the principal coordinates and correlated them with sample-level taxonomic profiles. Cluster 1 was characterized by a high relative abundance of *Lactobacillus* species, particularly *L. crispatus* and *L. iners*, and corresponded to *Lactobacillus*-dominant CSTs. Cluster 2 was driven by a high abundance of *Gardnerella vaginalis*, often accompanied by *Atopobium vaginae, Prevotella* spp., and other BV-associated taxa, corresponding to high-diversity CSTs. Cluster 3 was associated with *Streptococcus* spp. and other opportunistic pathogens (e.g., *Ureaplasma, Staphylococcus, Enterococcus*), representing a more heterogeneous group of non-canonical vaginal communities. This three-cluster structure recapitulates the fundamental ecological organization of the vaginal microbiome reported in previous studies (9, 11).

### CST distribution

**Fig 3:**
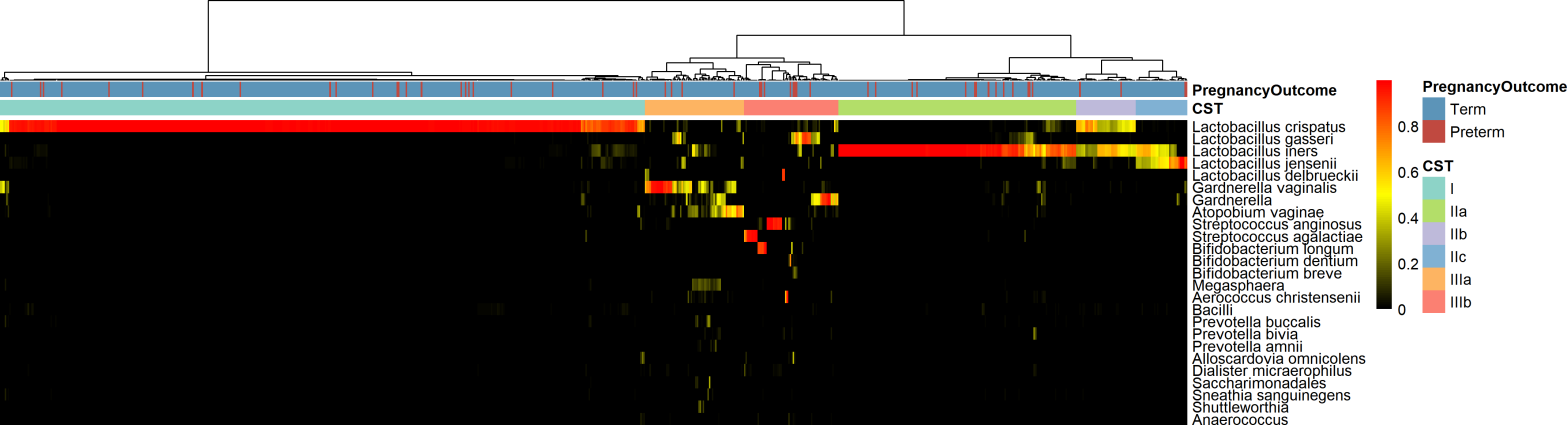
CST distribution. Bar plots showing CST distribution (enterotype-like clustering) across trimesters. Numbers above bars indicate sample sizes. CST I (dominated by atypical *Lactobacillus* subtypes), CST IIa (dominated by Lactobacillus iners), CST IIb (co-dominated by *L. iners* and atypical *Lactobacillus* subtypes), CST IIc (co-dominated by *L. iners* and *Lactobacillus jensenii*), CST IIIa (dominated by BV-associated taxa, including *Gardnerella vaginalis*, *Atopobium vaginae*, and *Megasphaera* spp.), CST IIIb (mixed community dominated by *Lactobacillus gasseri*, non-*G*. *vaginalis Gardnerella* subtypes, *Streptococcus anginosus*, *Streptococcus agalactiae*, and *Bifidobacterium longum*). Complete results in Table 2.

Our data-driven approach identified six CSTs with the following dominant taxa: CST I (dominated by *Lactobacillus crispatus*), CST IIa (dominated by *Lactobacillus iners*), CST IIb (co-dominated by *L. iners* and atypical *Lactobacillus* subtypes), CST IIc (co-dominated by *L. iners* and *Lactobacillus jensenii*), CST IIIa (dominated by BV-associated taxa, including *Gardnerella vaginalis*, *Atopobium vaginae*, and *Megasphaera* spp.), CST IIIb (mixed community dominated by *Lactobacillus gasseri*, non-*G*. *vaginalis Gardnerella* subtypes, *Streptococcus anginosus*, *Streptococcus agalactiae*, and *Bifidobacterium longum*) (Fig. 3). Table 2 presents the distribution of CSTs across trimesters and groups. Multivariable logistic regression adjusting for maternal age, BMI, and obstetric history revealed no significant association between any CST and sPTB risk after FDR correction (all *q* > 0.05; Table 2). This lack of association was consistent across all three trimesters. These findings indicate that static CST classification does not capture sPTB risk in this population.

**TABLE 2:** CST of samples from both cohorts and trimesters (data were given as *n*)^*a*^

	First trimester			Second trimester			Third trimester		
	Term	Preterm	*q* value^*b*^	Term	Preterm	*q* value^*b*^	Term	Preterm	*q* value^*b*^
*n*	235	21		246	24		243	10	
CST I	128	11	0.91	136	10	0.97	134	4	1
CST IIa	44	6	0.91	51	7	0.97	47	1	1
CST IIb	13	0	0.91	11	1	0.97	13	1	1
CST IIc	9	1	0.91	10	0	0.97	13	1	1
CST IIIa	21	1	0.94	20	2	0.97	20	1	1
CST IIIb	20	2	0.94	18	4	0.97	16	2	1

^*a*^ CST, community state type.

^*b*^ Multivariable logistic regression model to test for the association between CST versus pregnancy outcome, while controlling for maternal age, BMI, and history of adverse obstetric outcomes, such as sPTB, late miscarriage, and cervical insufficiency. The BH (Benjamini-Hochberg procedure [BH]) correction to obtain FDR-corrected *q* values from raw *P* values. Significant at *P* < 0.1.

**Fig 4:**
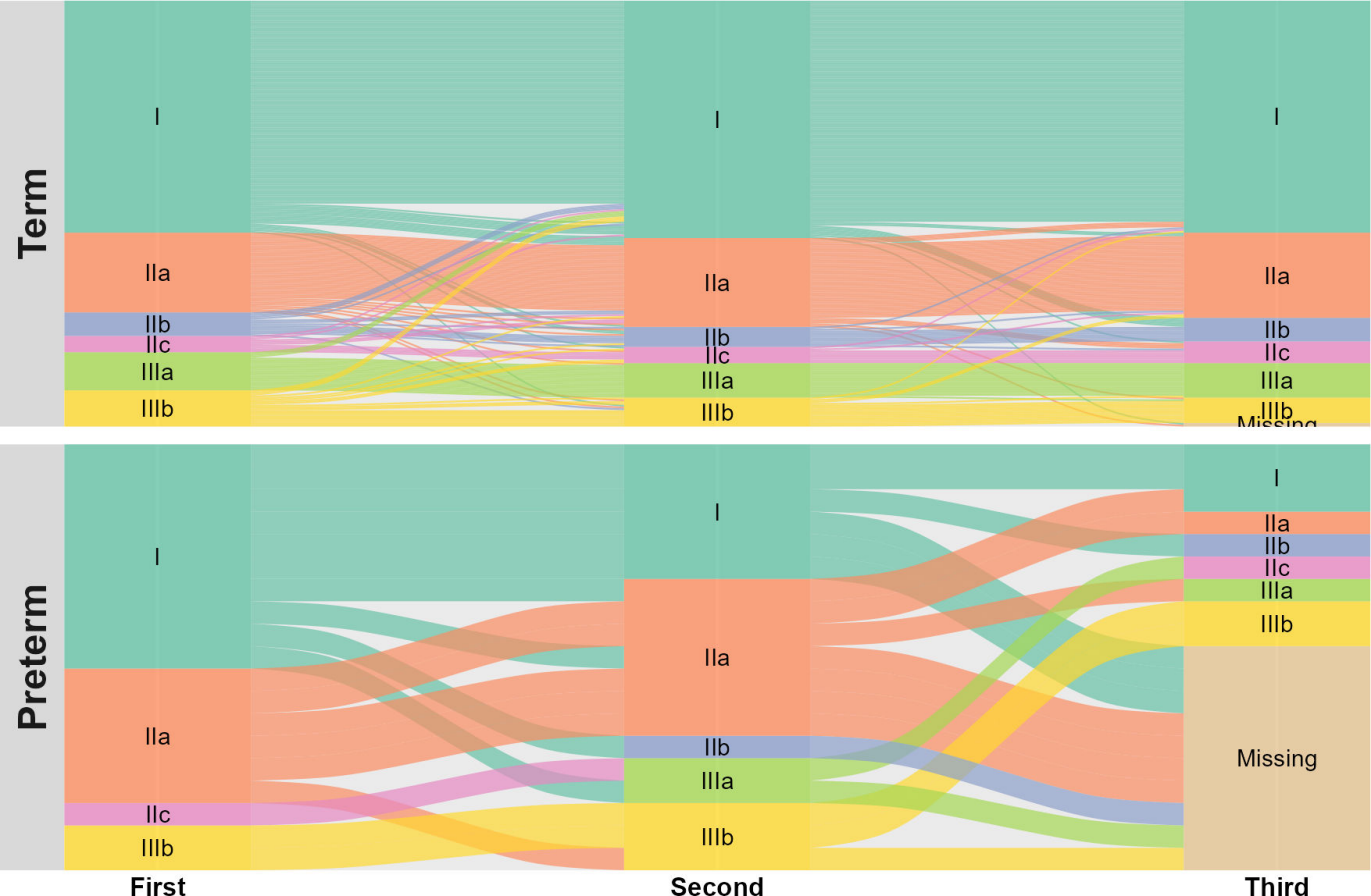
Longitudinal CST transitions (Sankey diagrams). Individual CST trajectories across trimesters for term (*n* = 247) and sPTB (*n* = 26). Bandwidth proportional to the number of participants. CST definitions as in Fig. 3. Term group maintained the same CST throughout pregnancy in 182 vs 5 in the sPTB group (*P* < 0.05, Fisher’s exact). “Missing” indicates samples unavailable due to preterm delivery before the third trimester.

While static CST distributions showed no association with sPTB, longitudinal analysis of CST transitions revealed certain differences in stability. Sankey diagrams (Fig. 4) illustrating CST trajectories across trimesters and statistical analysis demonstrated that 182 women in the term group maintained the same CST throughout pregnancy, compared to only 5 in the sPTB group (*P* < 0.05, Fisher’s exact test).

**Fig 5:**
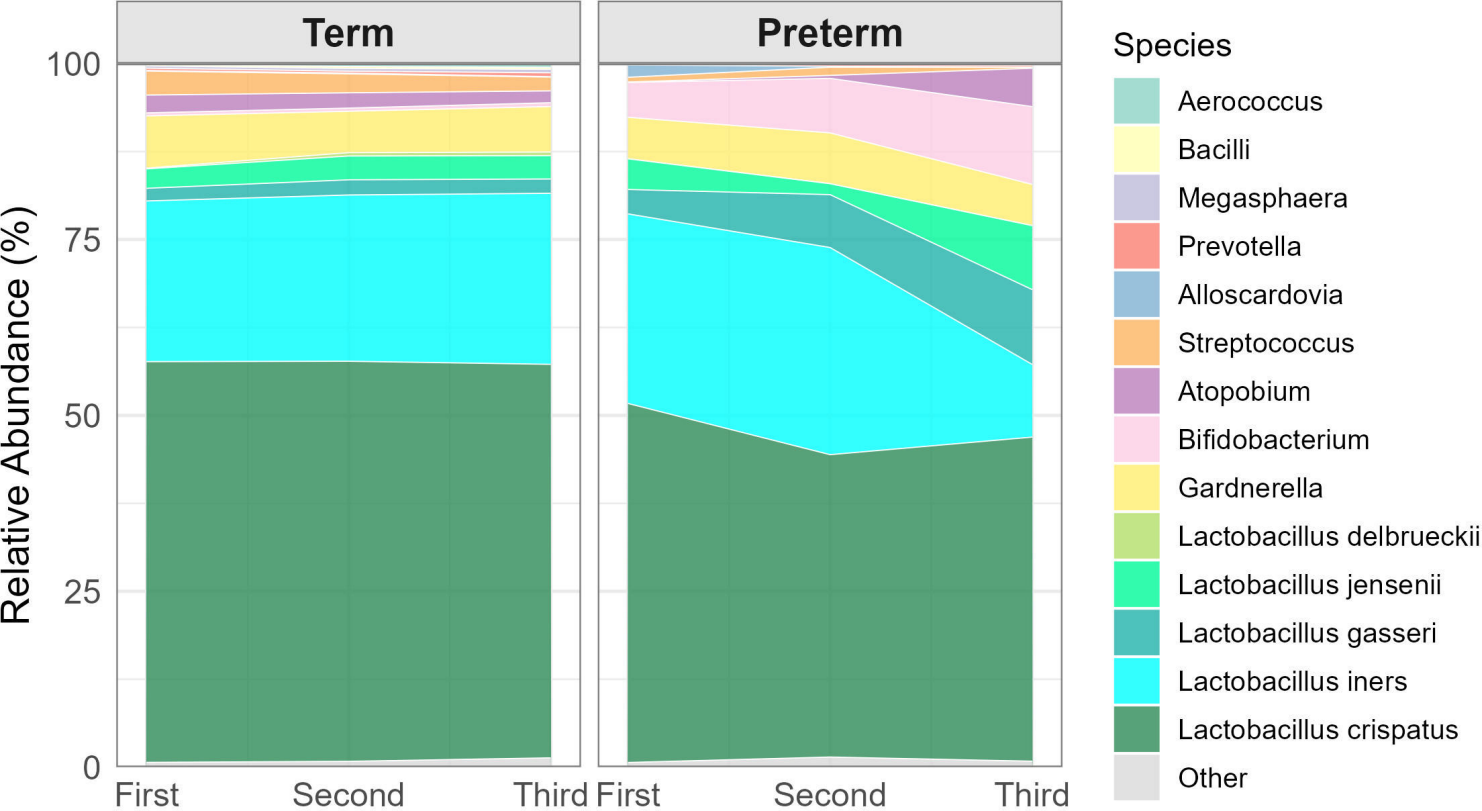
Stacked area graphs of vaginal community composition across three trimesters. Relative abundance of major bacterial genera across trimesters for the entire cohort. The *x*-axis shows trimesters (first, second, third), and the *y*-axis shows relative abundance (0%–100%). Term controls typically maintain stable dominance of a single *Lactobacillus* species (predominantly *L. crispatus* or *L. iners*) throughout pregnancy. sPTB cases frequently exhibit shifts between *Lactobacillus* dominance and communities with increased abundance of *Gardnerella*, *Atopobium*, or other anaerobic taxa, often with multiple transitions across trimesters.

**Fig 6:**
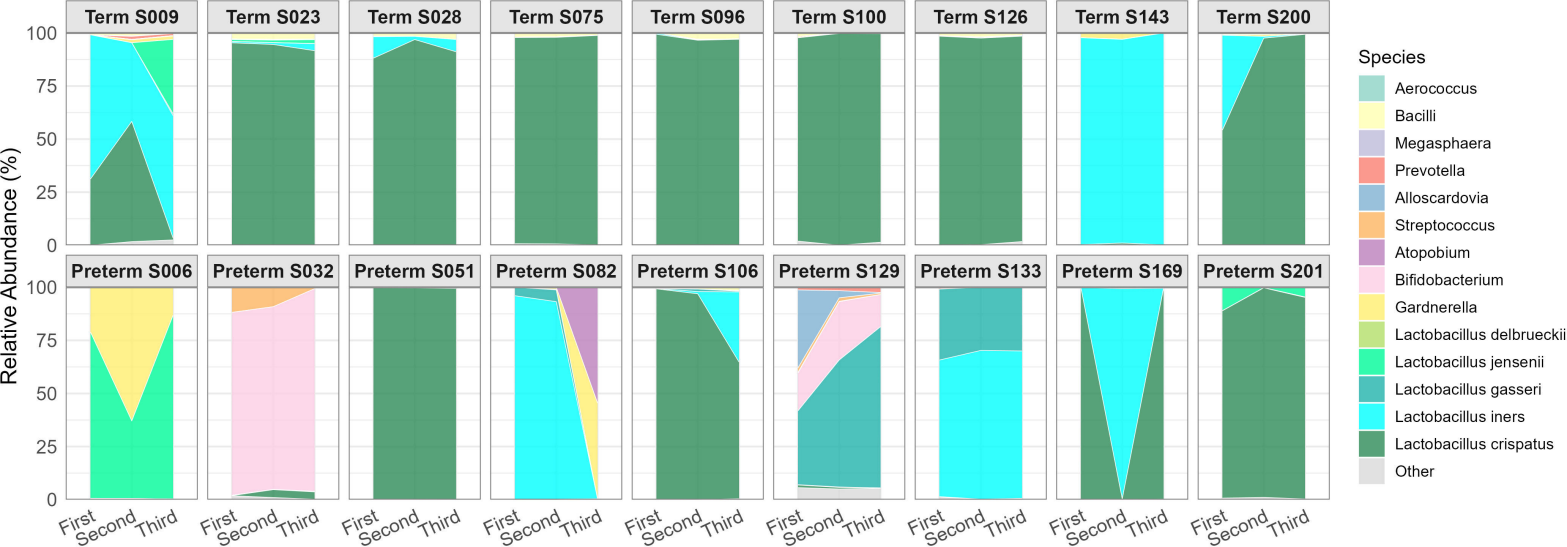
Individual-level stacked area graphs of community composition. Stacked area graphs showing the relative abundance of major bacterial genera across three trimesters for individual participants. All nine sPTB cases with complete longitudinal samples (first, second, and third trimesters). Nine randomly selected term controls matched for maternal age and BMI. Each plot represents one participant, with the x-axis showing trimesters and the y-axis showing relative abundance (0%–100%). Colors represent major bacterial genera as indicated in the legend. The plots illustrate marked compositional fluctuations in sPTB cases compared to relatively stable communities dominated by a single *Lactobacillus* species in term controls.

Stacked area graphs of community composition (Fig. 5 and 6) further illustrated this instability, showing marked compositional fluctuations in individual sPTB cases compared to relatively stable communities in term controls. Term controls typically maintain stable dominance of a single *Lactobacillus* species (predominantly *L. crispatus* or *L. iners*) throughout pregnancy. sPTB cases frequently exhibit shifts between *Lactobacillus* dominance and communities with increased abundance of *Gardnerella, Atopobium*, or *Bifidobacterium*, often with multiple transitions across trimesters (Fig. 5). All nine sPTB cases with complete longitudinal samples (first, second, and third trimesters) and nine randomly selected term controls matched for clinical characteristics were illustrated. The plots present marked compositional fluctuations in sPTB cases compared to relatively stable communities dominated by a single *Lactobacillus* species in term controls, indicating an increased microbial instability and harmful taxa in pregnancies resulting in sPTB (Fig. 6).

### Differential abundance analysis

**Fig 7:**
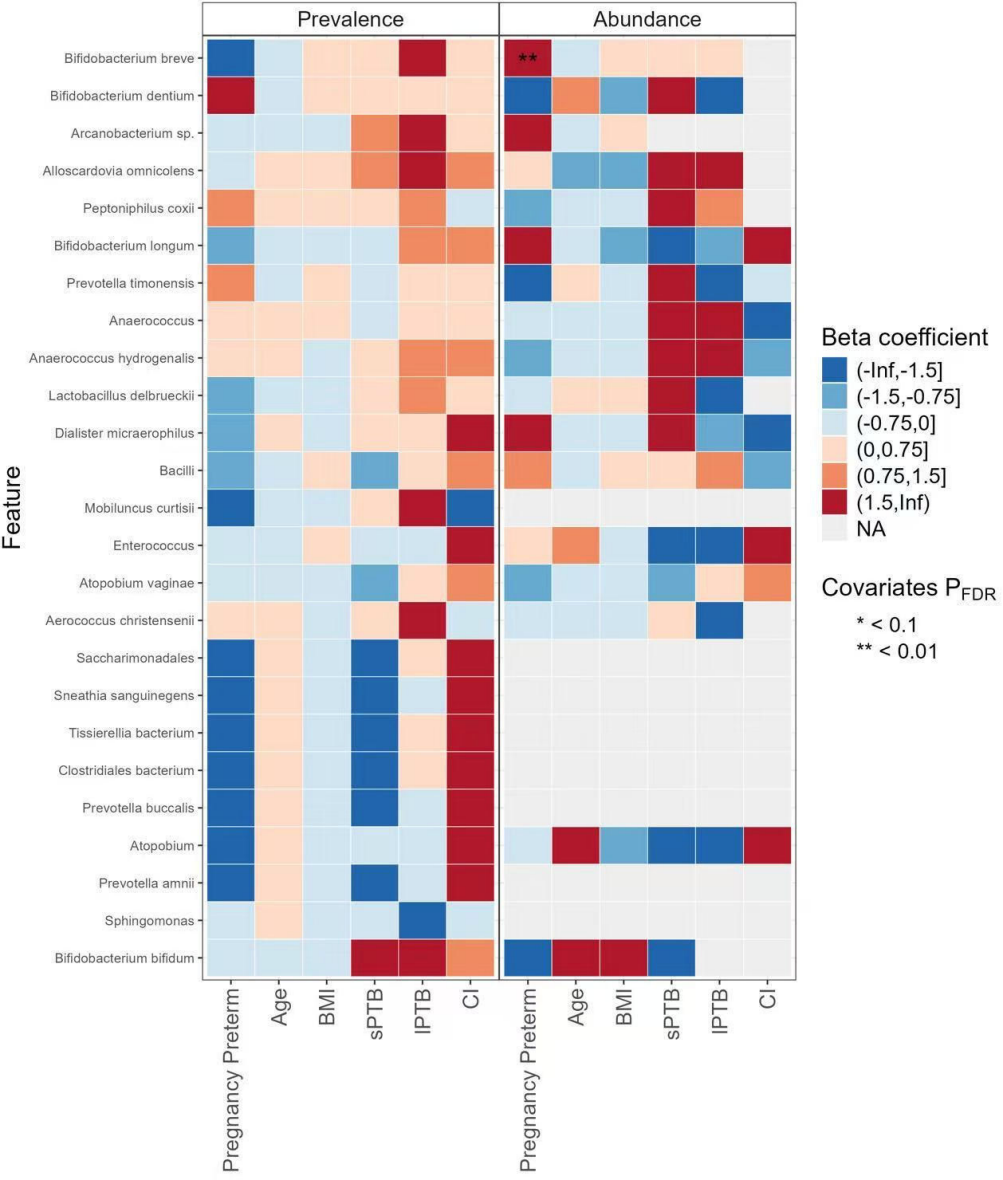
Differential abundance analysis. Heatmap showing taxa with nominally significant associations with sPTB in the second trimester (22–28 weeks) from MaAsLin3 analysis. The color scale indicates prevalence and abundance, with both set to 1.0 for all displayed taxa. *Bifidobacterium breve* is significantly overrepresented in the sPTB group, while no other taxa showed significant differences in the second trimester between the two groups after FDR correction; all the others had *q* values > 0.1 after multiple testing correction. *: *P* < 0.1. **: *P* < 0.01.

Differential abundance analysis using MaAsLin3 with covariate adjustment and FDR correction was performed on both 16S rRNA sequencing data and targeted qPCR data. Figure 7 presents taxa with differential abundance between groups identified by 16S rRNA sequencing. Except for *Bifidobacterium breve*, taxa showed no significant associations in the second trimester between the two groups after FDR correction; all the others had *q* values > 0.1 after multiple testing correction. Importantly, the significant taxa had very low prevalence and abundance (<1% relative abundance in most cases), limiting their biological and clinical significance.

Table 3 presents the relative abundance of specific bacterial targets measured by qPCR, and Table 4 presents prevalence data. Nominally significant associations (uncorrected *P* < 0.05) included *Ureaplasma urealyticum* abundance in the first trimester (*P* = 0.037), *Mycoplasma hominis* abundance in the second trimester (*P* = 0.030), *Bacteroides mobilis/Bacteroides fragilis* abundance in the second trimester (*P* = 0.030), and *Lactobacillus gasseri* prevalence in the third trimester (*P* = 0.028). However, none of these associations survived FDR correction, and several could not be reliably modeled due to extremely low detection frequencies (all *q* > 0.1). For example, *M. hominis* was detected in only 1/225 term and 1/20 sPTB samples in the second trimester, and *B. fragilis* in 1/225 term and 1/20 sPTB samples, making robust statistical inference impossible. Comparison of 16S and qPCR data revealed that *M. hominis* was not detected by 16S sequencing in any sample, whereas qPCR detected it in a small number of samples (*n* = 2 in the second trimester, *n* = 2 in the third trimester). *U. urealyticum* showed better concordance, with 16S detecting it in 9 samples and qPCR in 322 samples, including all 9 16S-positive samples.

**TABLE 3:** Relative abundance of specific bacterial targets in qPCR test (data were given as mean abundance from the samples)

	First trimester				Second trimester				Third trimester			
	Term	sPTB	*P* ^*a*^	*q* ^*b*^	Term	sPTB	*P* ^*a*^	*q* ^*b*^	Term	sPTB	*P* ^*a*^	*q* ^*b*^
*n*	232	21			225	20			220	9		
*Lactobacillus crispatus*	52.035	43.024	0.245	1	52.976	49.634	0.554	1	52.19	50.774	0.876	1
*Lactobacillus johnsonii*	5.545	3.116	0.716	1	6.361	0.524	0.478	1	3.908	0.661	0.864	1
*Lactobacillus gasseri*	1.486	4.168	0.183	1	2.148	8.621	0.242	1	2.145	14.629	0.005	1
*Lactobacillus iners*	21.44	27.879	0.673	1	23.612	15.913	0.882	1	23.951	22.694	0.364	1
BVAB1/2	0.062	0.005	0.449	1	0.385	0.047	0.988	NA^*c*^	0.088	0	0.288	NA
*Atopobium vaginalis*	2.203	2.096	0.556	1	2.056	0.305	0.522	NA	2.381	0.022	0.410	NA
*Gardnerella vaginalis*	10.537	10.636	0.965	1	6.879	11.402	0.187	1	8.092	0	0.048	NA
*BVAB-TM7*	0.078	0	0.774	NA	0.086	0	0.680	NA	0.063	0	0.787	NA
*Megacoccus*	0.007	0	0.391	NA	0.012	0	0.464	NA	0.025	0	0.497	NA
*Prevotella II*	0.098	0	0.335	NA	0.075	0	0.610	NA	0.445	0	0.656	NA
*Bacteroides mobilis/Bacteroides fragilis*	0.014	0	0.550	NA	0.004	0.068	0.030	NA	0.008	0	0.857	NA
*Sneathia amnii*	0.005	0	0.502	NA	0.023	0	0.610	NA	0.004	0	0.787	NA
*Candida*	0.319	0	0.123	NA	0.021	0.005	0.737	1	0.105	0	0.362	NA
*Candida krusei*	0	0	NA	NA	0	0	NA	NA	0.0000473	0	0.857	NA
*Candida glabrata*	0.01	0.007	0.580	NA	0.007	0.129	0.530	NA	0.007	0	0.692	NA
*Escherichia coli*	0.014	0	0.362	NA	0.459	0	0.395	NA	0.462	0.036	0.357	1
*Enterococcus faecalis*	0.216	0.161	0.641	NA	0.918	1.086	0.623	NA	1.573	0	0.239	NA
*Staphylococcus aureus*	0	0	NA	NA	0	0	NA	NA	0.005	0	0.787	NA
*Group B Streptococcus*	1.782	0.001	0.932	NA	1.323	0.001	0.973	NA	0.898	0	0.567	NA
*Ureaplasma urealyticum*	0.037	0.086	0.037	1	0.222	0.363	0.184	1	0.392	0.074	0.782	1
*Mycoplasma hominis*	0.001	0	0.774	NA	0.001	0.236	0.030	NA	0.011	0	0.787	NA
*Chlamydia trachomatis*	0.004	0	0.607	NA	0.003	0	0.680	NA	0.002	0	0.787	NA

^*a*^ Significant at *P* < 0.05. *P* values are uncorrected.

^*b*^ Use MaAsLin 3 to test for the association between specific bacterial targets versus pregnancy outcome, while controlling for maternal age, BMI, and history of adverse obstetric outcomes, such as sPTB, late miscarriage, and cervical insufficiency. The BH (Benjamini-Hochberg procedure [BH]) correction to obtain FDR-corrected *q* values from raw *P* values. Significant at *P* < 0.1.

^*c*^ NA, not applicable.

**TABLE 4:** Detection of specific bacterial targets in qPCR test (data were given as *n* [%])

	First trimester				Second trimester				Third trimester			
	Term	sPTB	*P* ^*a*^	*q* ^*b*^	Term	sPTB	*P* ^*a*^	*q* ^*b*^	Term	sPTB	*P* ^*a*^	*q* ^*b*^
*n*	232	21			225	20			220	9		
*Lactobacillus crispatus*	155 (66.8)	11 (52.4)	0.183	1	160 (71.1)	12 (60.0)	0.298	1	153 (69.5)	6 (66.7)	1.000	1
*Lactobacillus johnsonii*	73 (31.5)	6 (28.6)	0.784	1	74 (32.9)	6 (30.0)	0.792	1	40 (18.2)	2 (22.2)	1.000	1
*Lactobacillus gasseri*	23 (9.9)	4 (19.0)	0.353	1	26 (11.6)	4 (20.0)	0.454	1	28 (12.7)	4 (44.4)	0.028	1
*Lactobacillus iners*	155 (66.8)	15 (71.4)	0.666	1	147 (65.3)	14 (70.0)	0.673	1	145 (65.9)	4 (44.4)	0.333	1
BVAB1/2	23 (9.9)	1 (4.8)	0.702	1	24 (10.7)	2 (10.0)	1.000	1	25 (11.4)	0	0.602	1
*Atopobium vaginalis*	48 (20.7)	3 (14.3)	0.677	1	50 (22.2)	3 (15.0)	0.640	1	52 (23.6)	1 (11.1)	0.638	1
*Gardnerella vaginalis*	97 (41.8)	9 (42.9)	0.926	1	70 (31.1)	9 (45.0)	0.203	1	70 (31.8)	0	0.060	1
BVAB-TM7	1 (0.4)	0	1.000	NA^*c*^	2 (0.9)	0	1.000	1	2 (0.9)	0	1.000	1
*Megacoccus*	8 (3.4)	0	1.000	1	6 (2.7)	0	1.000	1	11 (5.0)	0	1.000	1
*Prevotella II*	10 (4.3)	0	1.000	1	3 (1.3)	0	1.000	1	5 (2.3)	0	1.000	1
*Bacteroides mobilis/Bacteroides fragilis*	4 (1.7)	0	1.000	1	1 (0.4)	1 (5.0)	0.383	1	1 (0.45)	0	1.000	NA
*Sneathia amnii*	5 (2.2)	0	1.000	1	3 (1.3)	0	1.000	1	2 (0.9)	0	1.000	1
*Candida*	24 (10.3)	0	0.236	1	16 (7.1)	1 (5.0)	1.000	1	19 (8.6)	0	1.000	1
*Candida krusei*	0	0	1.000	NA	0	0	1.000	NA	1 (0.45)	0	1.000	NA
*Candida glabrata*	6 (2.6)	1 (4.8)	1.000	1	6 (2.7)	1 (5.0)	1.000	1	4 (1.8)	0	1.000	1
*Escherichia coli*	9 (3.9)	0	1.000	1	8 (3.6)	0	1.000	1	10 (4.5)	1 (11.1)	0.914	1
*Enterococcus faecalis*	18 (7.8)	1 (4.8)	0.947	1	16 (7.1)	2 (10.0)	0.978	1	30 (13.6)	0	0.610	1
*Staphylococcus aureus*	0	0	1.000	NA	0	0	1.000	NA	2 (0.9)	0	1.000	1
*Group B Streptococcus*	12 (5.2)	1 (4.8)	1.000	1	12 (5.3)	1 (5.0)	1.000	1	8 (3.6)	0	1.000	1
*Ureaplasma urealyticum*	95 (40.9)	12 (57.1)	0.150	1	98 (43.6)	11 (55.0)	0.324	1	103 (46.8)	3 (33.3)	0.650	1
*Mycoplasma hominis*	1 (0.4)	0	1.000	NA	1 (0.4)	1 (5.0)	0.383	1	2 (0.9)	0	1.000	1
*Chlamydia trachomatis*	3 (1.3)	0	1.000	1	2 (0.9)	0	1.000	1	2 (0.9)	0	1.000	1

^*a*^ Significant at *P* < 0.05. *P* values are uncorrected.

^*b*^ Use MaAsLin 3 to test for the association between specific bacterial targets versus pregnancy outcome, while controlling for maternal age, BMI, and history of adverse obstetric outcomes, such as sPTB, late miscarriage, and cervical insufficiency. The BH (Benjamini-Hochberg Procedure [BH]) correction to obtain FDR-corrected *q* values from raw *P* values. Significant at *P* < 0.1.

^*c*^ NA, not applicable.

## DISCUSSION

Our study yields two primary findings. First, while we found no significant association between any specific CST and sPTB risk after rigorous multivariable adjustment and multiple testing correction, we observed that longitudinal CST instability across pregnancy was significantly associated with sPTB. Second, several taxa, including *Ureaplasma urealyticum*, *Mycoplasma hominis*, and *Bacteroides* species, showed nominally significant associations with sPTB in univariate analyses. However, none of these associations survived FDR correction, largely due to their very low prevalence and abundance. Despite failing to reach statistical significance after correction, the consistent detection of these organisms, almost exclusively in sPTB cases, raises the possibility that even low-abundance pathobionts may contribute to sPTB pathogenesis.

Our inability to replicate the CST-sPTB association observed in some Western cohorts (11) aligns with other studies reporting null results (12, 13), firmly placing our work within a critical scientific discourse on the generalizability of microbial risk models. The CST framework, while useful for broad ecological categorization, is likely too coarse to capture functionally significant, fine-scale variations that drive pathology (23, 24). Our results indicate that the predictive value of vaginal microbial features is population-context-dependent, likely shaped by host genetics, lifestyle, and environmental exposures (25–28). Therefore, risk prediction models developed in one ethnic group cannot be directly extrapolated to others without validation. Critically, our study addresses a significant gap in the literature by providing the first longitudinal characterization of the vaginal microbiome in relation to sPTB in a Chinese cohort.

Beyond static classification, our longitudinal design revealed that microbial instability itself is an important risk indicator. The significantly lower rate of CST persistence in the sPTB group suggests that an inability to maintain a stable ecological state may be more pathogenic than residing in any single state. This finding is consistent with the concept that pregnancy requires a stable, *Lactobacillus*-dominated environment to maintain cervical integrity and prevent ascending infection (5, 7). This dynamic perspective represents a crucial advance over snapshot analyses and has important implications for clinical risk prediction. Communities that frequently shift between states may create intermittent windows of vulnerability; even if no single state is intrinsically pathogenic, monitoring changes in community composition over time may prove more informative than single-time-point assessments.

The three-cluster structure observed in our PCoA and NMDS plots, comprising *Lactobacillus*-dominant, *Gardnerella*-dominant, and *Streptococcus*-dominated communities, reflects the well-established ecological organization of the vaginal microbiome (9, 11). The primary axis of variation separates *Lactobacillus*-dominant states from *Lactobacillus*-deficient states, consistent with the concept that depletion of protective lactobacilli creates a niche for colonization by diverse anaerobic and facultative bacteria. The secondary separation between *Gardnerella*-dominated and *Streptococcus*/other-dominated communities within the *Lactobacillus*-deficient spectrum suggests that multiple distinct dysbiotic states exist, potentially with different functional properties and clinical implications. Notably, despite the clear separation of samples into these three clusters, we found no significant association between cluster membership and sPTB risk at any individual time point. This finding aligns with the growing recognition that static taxonomic classifications, while useful for ecological description, may have limited predictive value for adverse pregnancy outcomes (13, 14).

Furthermore, our species-level qPCR data revealed the role of specific, potentially interactive pathobionts. The association of *U. urealyticum* and *M. hominis* with sPTB is consistent with their known pro-inflammatory potential (29–33). However, their high prevalence in healthy women (29) suggests they are necessary but not sufficient causes, acting perhaps in concert with other community members or host factors. In our study, no individual taxon remained significantly associated with sPTB after rigorous FDR correction. Several factors may explain this discrepancy: First, limited statistical power due to the modest sPTB sample size (*n* = 26) likely contributed to the failure of nominally significant associations to survive multiple testing correction. With 30+ taxa tested across multiple time points, our sample size was insufficient to overcome the FDR correction. Second, low prevalence of putative pathobionts, such as *M. hominis* and *B. fragilis* (<1% in most samples), meant that even if these organisms contribute to sPTB risk, detecting their effect requires substantially larger cohorts. The fact that these organisms were detected almost exclusively in sPTB cases is intriguing and warrants further investigation. Third, methodological limitations of 16S sequencing, including the inability to resolve species and strains, may obscure true associations. For example, *G. vaginalis* pathogenicity is known to be clade-specific (34–36), and our inability to distinguish pathogenic from commensal subtypes likely diluted any true signal. Similarly, distinguishing *Ureaplasma parvum* from *U. urealyticum* is crucial, as studies implicate *U. parvum* more strongly in sPTB (37–40).

The discrepancy between 16S and qPCR detection of low-abundance organisms like *M. hominis* highlights the complementary value of targeted molecular approaches. 16S sequencing failed to detect *M. hominis* in any sample, likely due to its low abundance falling below the sequencing depth detection threshold, while qPCR successfully identified it in a small number of samples. This suggests that targeted approaches may be necessary to detect rare but potentially pathogenic organisms, and that 16S-based studies may underestimate the role of low-abundance taxa. However, the extremely low detection frequency also means that even qPCR may lack sensitivity for population-level studies unless sample sizes are very large. Future studies should consider enrichment strategies or deeper sequencing to capture rare taxa.

This study has several notable strengths, including its prospective longitudinal design in an understudied Chinese cohort, the inclusion of both low- and high-risk pregnancies, and the application of complementary molecular detection methods (qPCR and 16S sequencing) across three trimesters. However, several limitations must be acknowledged. The modest sample size in the sPTB group limited our statistical power and likely contributed to the failure of nominally significant associations to survive multiple testing correction. This is particularly problematic for detecting associations with low-prevalence taxa, where even large effect sizes may be missed. Another methodological consideration inherent to longitudinal studies of PTB is the progressive reduction in sample size in the sPTB group across trimesters. In our cohort, the number of sPTB samples decreased to 10 in the third trimester, reflecting the fact that women delivering before 34 weeks (53.8% of sPTB cases) could not contribute later samples. Consequently, our third-trimester findings are specific to the late preterm subgroup (34–36 weeks) and may not generalize to early PTB. However, our primary conclusions regarding microbial stability and early-pregnancy taxonomic associations are based on larger first- and second-trimester samples, which are most relevant for early risk prediction. Future studies with larger, multi-center cohorts should aim to enroll sufficient numbers of early and late preterm cases to enable stratified analyses by gestational age at delivery. Methodologically, while 16S rRNA and qPCR are powerful tools, they offer limited phylogenetic and functional resolution. They cannot reliably distinguish between closely related species or serovars or provide direct insight into microbial gene expression, metabolic output, or host immune interactions. The absence of placental or amniotic samples also precluded direct confirmation of microbial ascent and its role in the sPTB cases. Additionally, while we adjusted for multiple potential confounders, residual confounding from unmeasured factors cannot be excluded. Future research should prioritize larger, multi-center cohorts to enhance statistical power and generalizability. Employing shotgun metagenomics will be crucial to achieve species- and strain-level resolution, enabling the identification of specific pathogenic clades. Integrating metatranscriptomics, metabolomics, and host immune profiling will be essential to move beyond correlation and elucidate the functional pathways and mechanisms through which the vaginal microbiome contributes to sPTB.

### Conclusion

In this Chinese Han cohort, sPTB was not associated with static CST classification but was significantly linked to reduced temporal stability of the vaginal microbiome CST across pregnancy. Several taxa (*Ureaplasma urealyticum, Mycoplasma hominis, Bacteroides* spp.) showed nominally significant associations but did not survive FDR correction, which may be due to very low abundance. These findings suggest that ecological instability may be an indicator of sPTB risk in this population, and the microbial etiology of sPTB is complex, context-dependent, and likely population-specific, involving intricate inter-taxa interactions and host-microbe dynamics that cannot be reduced to single biomarkers or broad community types. Future predictive models must incorporate these dynamic and fine-scale features to achieve clinical utility.

## Supplementary Material

Reviewer comments

## Data Availability

The data sets used and analyzed during the current study are available from the China National Center for Bioinformation (CNCB), under GSA: CRA037472.

## SUPPLEMENTAL MATERIAL

OPEN PEER REVIEW reviewer-comments.pdf An accounting of the reviewer comments and feedback.

ASM does not own the copyrights to Supplemental Material that may be linked to, or accessed through, an article. The authors have granted ASM a non-exclusive, world-wide license to publish the Supplemental Material files. Please contact the corresponding author directly for reuse.
