# Supplementary material for: Longitudinal changes in the vaginal microbiome associate with spontaneous preterm birth: a focus on stability and specific taxa in a Chinese cohort
Source: Microbiol Spectr. 2026 Jul 17;14(8):e03705-25. doi: 10.1128/spectrum.03705-25 (PMC13435760; doi:10.1128/spectrum.03705-25)
Supplement: Supplementary file 1 [file reviewer-comments.pdf]

AMERICAN SOCIETY FOR MICROBIOLOGY | Microbiology Spectrum

Longitudinal Changes in the Vaginal Microbiome Associate with Spontaneous Preterm Birth: A Focus on Stability and Specific Taxa in a Chinese Cohort

Yi Yu, Jingwen Zhou, Qian Zhou, Xu Li, Lanying Zhang, Sabrina Li, Baoli Zhu, Jinsong Gao, and Juntao Liu

Corresponding Author(s): Juntao Liu, Peking Union Medical College Hospital Department of Obstetrics and Gynecology

Review Timeline:

Submission Date:	November 15, 2025
Editorial Decision:	January 12, 2026
Revision Received:	March 3, 2026
Editorial Decision:	April 2, 2026
Revision Received:	April 10, 2026
Accepted:	April 14, 2026

Editor: Zhe Li

Reviewer(s): Disclosure of reviewer identity is with reference to reviewer comments included in decision letter(s). The following individuals involved in review of your submission have agreed to reveal their identity: Shengru Wu (Reviewer #1)

Transaction Report:

(Note: With the exception of the correction of typographical or spelling errors that could be a source of ambiguity, letters and reports are not edited. The original formatting of letters and referee reports may not be reflected in this compilation.)

DOI: https://doi.org/10.1128/spectrum.03705-25

Re: Spectrum03705-25 (**Longitudinal Changes in the Vaginal Microbiome Associate with Spontaneous Preterm Birth: A Focus on Stability and Specific Taxa in a Chinese Han Cohort**)

Dear Dr. Juntao Liu:

Thank you for the privilege of reviewing your work. Below you will find my comments, instructions from the Spectrum editorial office, and the reviewer comments.

Please return the manuscript within 60 days; if you cannot complete the modification within this time period, please contact me. If you do not wish to modify the manuscript and prefer to submit it to another journal, notify me immediately so that the manuscript may be formally withdrawn from consideration by Spectrum.

Revision Guidelines
To submit your modified manuscript, log into the submission site at https://spectrum.msubmit.net/cgi-bin/main.plex. Go to Author Tasks and click the appropriate manuscript title to begin. The information you entered when you first submitted the paper will be displayed; update this as necessary. Note the following requirements:

• Upload point-by-point responses to the issues raised by the reviewers in a file named "Response to Reviewers," NOT in your cover letter.
• Upload a compare copy of the manuscript (without figures) as a "Marked-Up Manuscript" file.
• Upload a clean .DOC/.DOCX version of the revised manuscript and remove the previous version.
• Each figure must be uploaded as a separate, editable, high-resolution file (TIFF or EPS preferred), and any multipanel figures must be assembled into one file.
• Any supplemental material intended for posting by ASM should be uploaded with their legends separate from the main manuscript. You can combine all supplemental material into one file (preferred) or split it into a maximum of 10 files with all associated legends included.

For complete guidelines on revision requirements, see our Submission and Review Process webpage. Submission of a paper that does not conform to guidelines may delay acceptance of your manuscript.

Data availability: ASM policy requires that data be available to the public upon online posting of the article, so please verify all links to sequence records, if present, and make sure that each number retrieves the full record of the data. If a new accession number is not linked or a link is broken, provide Spectrum production staff with the correct URL for the record. If the accession numbers for new data are not publicly accessible before the expected online posting of the article, publication may be delayed; please contact production staff (Spectrum@asmusa.org) immediately with the expected release date.

Publication Fees: For information on publication fees and which article types are subject to charges, visit our website. If your manuscript is accepted for publication and any fees apply, you will be contacted separately about payment during the production process; please follow the instructions in that e-mail. Arrangements for payment must be made before your article is published.

ASM Membership: Corresponding authors may join or renew ASM membership to obtain discounts on publication fees. Need to upgrade your membership level? Please contact Customer Service at Service@asmusa.org.

The ASM Journals program strives for constant improvement in our submission and publication process. Please tell us how we can improve your experience by taking this quick Author Survey.

Thank you for submitting your paper to Spectrum.

Sincerely,
Zhe Li
Editor
Microbiology Spectrum

Reviewer #1 (Comments for the Author):

My detail comments for the present manuscript were listed as below:
(1) Although the current Introduction concludes by mentioning the aim to "move beyond the CST framework," it does not explicitly state testable research hypotheses (e.g., "We hypothesize that sPTB is associated with reduced temporal stability of the vaginal microbiome and enrichment of specific pathobionts, rather than CST type per se"). We recommend clearly listing 1-2

core hypotheses in the final paragraph to enhance the focus and clarity of the study design.

(2) The authors are encouraged to cite prior studies on the vaginal microbiome in Asian or Chinese populations (if available) in the Introduction to justify why validating Western-derived microbial models in this specific cohort is particularly necessary. This would better highlight the novelty and gap-filling value of the present work.

(3) In the differential abundance analyses, multiple statistical tests were performed across numerous taxa and time points, yet the manuscript does not mention whether corrections for multiple comparisons (e.g., FDR or Bonferroni) were applied. This omission may inflate the false discovery rate. Please clarify the correction strategy used, or explicitly label uncorrected P-values as such when interpreting results.

(4) CST classification itself remains a contentious approach in the field. We suggest that the authors complement their CST analysis with enterotype-like clustering (or alternative community stratification methods) tailored to their own dataset, and report the resulting distribution patterns to provide a more robust ecological characterization.

(5) The Results section indicates significant differences between the sPTB and term groups in maternal age, BMI, and history of adverse obstetric outcomes. However, the primary analyses (e.g., CST stability and specific taxon abundance) appear not to have been adjusted for these potential confounders via multivariable regression. We strongly recommend incorporating covariate-adjusted models to strengthen causal inference.

(6) The quality and informativeness of the figures in this manuscript are suboptimal. We encourage the authors to include more customized or advanced visualizations (e.g., longitudinal trajectory plots, network analyses, or effect size displays) to extract and communicate deeper biological insights from their rich dataset.

Reviewer #2 (Comments for the Author):

I enjoyed reading the study submitted by Yu and colleagues and applaud their collection of a valuable longitudinal dataset. I have the following comments which must be addressed:

Major issues
1. There is no explicit statement of: 1) ethical approval being granted for this study; 2) informed consent being sought from patients; and 3) the sequencing data being deposited in a publicly available repository. I am assuming that this was an accidental oversight by the authors, but all three MUST be provided. This is a requirement of Microbiology Spectrum.

2. The authors have chosen to take an OTU clustering approach (in QIIME1) rather than a more modern approach of using amplicon sequence variants (using QIIME2), which isn't inherently disqualifying, but their method for taxonomic assignment is contradictory. Line 118 states that they used the GreenGenes database (which hasn't been updated since 2013) but line 121 states that taxonmy was assigned via BLAST search against the "NCBI 16S rRNA" database. Which method was actually used, and with which version of the database? Was the NCBI database they refer to the one available at: https://ftp.ncbi.nlm.nih.gov/refseq/TargetedLoci/Bacteria/ ? These details need to be added to the methods. The authors should also consider the use of older approaches in their future work. Species level resolution with v3/v4 sequences is difficult, even more so when clustering at 97 % similarity (https://doi.org/10.1093/bioinformatics/bty113; essentially, the 97% threshold is outdated and too low for assigning species).

3. The differential abundance analysis performed by the analysis is incredibly simplistic; a comparison of raw relative abundances (without adjusting for multiple comparisons!) is not appropriate when a multitude of dedicated tools for determining differential abundance exist. The authors must conduct a formal differential abundance analysis using purpose-built tools (e.g. ANCOM-BC, MaAsLin3, ALDEx2). I will leave the choice of tool up to the authors but reiterate that their current analysis is insufficient.

4. The authors do not give any detail as to how the qPCR data in Table 3 has been processed. Table 3 shows the relative abundance of the species as determined by qPCR but there is no explanation in the methods section as to how this was calculated. Proportional abundance of the summed copy number per mL? This needs to be stated clearly in the methods and in the table legend. Moreover, the data in Tables 3 and 4 are not discussed chronologically. The authors go back and forth discussing data from each table. Please re-write lines 177 - 185 so that all the observations from Table 3 are discussed before those from Table 4.

5. The language used by the authors to describe some of their findings is far too strong and 'oversells' their data. For example, these P values in lines 157-158 regarding alpha diversity metrics in Figure 1 are nowhere close to a 'trend'. A far more accurate statement is, "there was no significant difference between term and sPTB groups in terms of alpha diversity by any metric tested", which is what their data shows. Likewise, ANOSIM may well be returning a significant P value, but the NMDS and PCoA plots show a complete overlap of the term and sPTB groups at every trimester. All of the plots in Figure 2A and 2B (why are these on separate pages?) show a clear separation of the samples into three main groups, which is not even discussed. What is

the driver of this separation? Presumably it corresponds to lacto-dominant vs. lacto-deficient samples? I would like to see this discussed- perhaps labels showing the loadings of the taxa could be added (i.e. a compositional biplot).

6. Similar to the point above, I do not think that the way that the authors have presented the beta-diversity data (as PCA/nMDS plots) shows 'instability' as one cannot see the samples from the same patient across time on the same plot. Instability would suggest that the community is constantly changing. Longitudinal data is very valuable, and the authors have gone to great lengths to collect these samples, yet they are not making the most of it at present. To make any comment about instability, the authors could (and should) create stacked area graphs of the community composition (e.g. relative abundance) over the three trimesters they have measured. This could be done in R quite easily using ggplot2, and a separate plot produced for each of the 26 sPTB samples (e.g. using ggplot's faceting feature). See https://r-graph-gallery.com/136-stacked-area-chart.html for an example. A similar figure could be produced for 26 random term samples for comparison.

7. Where the authors do explicitly mention stability, the data presented is limited. This data is important and should be presented in the paper. I ask that the authors create two sankey diagrams (or some other visualisation) showing the change in CST assignments of each individual across the three trimesters, one for the term group and another for the sPTB group. See https://r-graph-gallery.com/sankey-diagram.html for examples.

Minor issues
1. Line 57: was intra-amniotic infection documented in any of the 26 PTB cases? If so, was any culture attempted? Causative organisms? This would be interesting to know and to see if the 16S or qPCR data corroborate this.

2. Line 112: which kit was used for library preparation?

3. Lines 148-149: Have the 17 P values in Table 1 been adjusted for multiple testing? Many differences between groups are relatively small with a large standard deviation e.g. BMI of 22.1 vs 21.4 both with a standard deviation of ~3.

4. Lines 164-165: The statement "This distinction was visually corroborated by NMDS (Figure 2A) and PCoA (Figure 2B) plots" is not accurate at all- readers cannot even tell which data points come from the same sample, so how can there be any judgement of stability? Please delete.

5. Figure 2: The large number of samples make it difficult to see the individual data points. Could the colours be made semi-transparent to show where multiple data points overlap? It also appears that there are only 8 samples in the 3rd trimester beta diversity plots. I assume this is also due to overlapping data points?

6. Line 166: "As defined in the methods," is unnecessary.

7. Figure 3: I found it quite difficult to distinguish between the white page background and the low relative abundance values in the heatmap. I would ask the authors to consider changing the colour scale so that the colour scale goes from black, to yellow, to red, as abundance increases.

8. Lines 210-212: The finding regarding Mycoplasma hominis is something I don't disbelieve, as it has been reported by several other groups, but Table 4 and Figure 4 shows that the number of samples with detectable M. hominis is incredibly small and is in some cases 1 vs. 0. This limitation needs to be discussed. How do the 16S and qPCR data for M. hominis (and U. urealyticum) line up?

<u>Review of Yu *et al*. 2026</u>

I enjoyed reading the study submitted by Yu and colleagues and applaud their collection of a valuable longitudinal dataset. I have the following issues which must be addressed:

Major issues

1. There is no explicit statement of: 1) ethical approval being granted for this study; 2) informed consent being sought from patients; and 3) the sequencing data being deposited in a publicly available repository. I am assuming that this was an accidental oversight by the authors, but all three MUST be provided. This is a requirement of Microbiology Spectrum.

2. The authors have chosen to take an OTU clustering approach (in QIIME1) rather than a more modern approach of using amplicon sequence variants (using QIIME2), which isn't inherently disqualifying, but their method for taxonomic assignment is contradictory. Line 118 states that they used the GreenGenes database (which hasn't been updated since 2013) but line 121 states that taxonmy was assigned via BLAST search against the "NCBI 16S rRNA" database. Which method was actually used, and with which version of the database? Was the NCBI database they refer to the one available at: https://ftp.ncbi.nlm.nih.gov/refseq/TargetedLoci/Bacteria/ ? These details need to be added to the methods. The authors should also consider the use of older approaches in their future work. Species level resolution with v3/v4 sequences is difficult, even more so when clustering at 97 % similarity (https://doi.org/10.1093/bioinformatics/bty113; essentially, the 97% threshold is outdated and too low for assigning species).

3. The differential abundance analysis performed by the analysis is incredibly simplistic; a comparison of raw relative abundances (without adjusting for multiple comparisons!) is not appropriate when a multitude of dedicated tools for determining differential abundance exist. The authors ***must*** conduct a formal differential abundance analysis using purpose-built tools (e.g. ANCOM-BC, MaAsLin3, ALDEx2). I will leave the choice of tool up to the authors but reiterate that their current analysis is insufficient.

4. The authors do not give any detail as to how the qPCR data in Table 3 has been processed. Table 3 shows the relative abundance of the species as determined by qPCR but there is no explanation in the methods section as to how this was calculated. Proportional abundance of the summed copy number per mL? This needs to be stated clearly in the methods and in the table legend. Moreover, the data in Tables 3 and 4 are not discussed chronologically. The authors go back and forth discussing data from each table. Please re-write lines 177 – 185 so that all the observations from Table 3 are discussed before those from Table 4.

5. The language used by the authors to describe some of their findings is far too strong and 'oversells' their data. For example, these *P* values in lines 157-158 regarding alpha

diversity metrics in Figure 1 are nowhere close to a 'trend'. A far more accurate statement is, "there was no significant difference between term and sPTB groups in terms of alpha diversity by any metric tested", which is what their data shows. Likewise, ANOSIM may well be returning a significant *P* value, but the NMDS and PCoA plots show a complete overlap of the term and sPTB groups at every trimester. All of the plots in Figure 2A and 2B (why are these on separate pages?) show a clear separation of the samples into three main groups, which is not even discussed. What is the driver of this separation? Presumably it corresponds to lacto-dominant vs. lacto-deficient samples? I would like to see this discussed- perhaps labels showing the loadings of the taxa could be added (i.e. a compositional biplot).

6. Similar to the point above, I do not think that the way that the authors have presented the beta-diversity data (as PCA/nMDS plots) shows 'instability' as one cannot see the samples from the same patient across time on the same plot. Instability would suggest that the community is constantly changing. Longitudinal data is very valuable, and the authors have gone to great lengths to collect these samples, yet they are not making the most of it at present. To make any comment about instability, the authors could (and should) create stacked area graphs of the community composition (e.g. relative abundance) over the three trimesters they have measured. This could be done in R quite easily using ggplot2, and a separate plot produced for each of the 26 sPTB samples (e.g. using ggplot's faceting feature). See https://r-graph-gallery.com/136-stacked-area-chart.html for an example. A similar figure could be produced for 26 random term samples for comparison.

7. Where the authors do explicitly mention stability, the data presented is limited. This data is important and should be presented in the paper. I ask that the authors create two sankey diagrams (or some other visualisation) showing the change in CST assignments of each individual across the three trimesters, one for the term group and another for the sPTB group. See https://r-graph-gallery.com/sankey-diagram.html for examples.

Minor issues

1. Line 57: was intra-amniotic infection documented in any of the 26 PTB cases? If so, was any culture attempted? Causative organisms? This would be interesting to know and to see if the 16S or qPCR data corroborate this.

2. Line 112: which kit was used for library preparation?

3. Lines 148-149: Have the 17 *P* values in Table 1 been adjusted for multiple testing? Many differences between groups are relatively small with a large standard deviation e.g. BMI of 22.1 vs 21.4 both with a standard deviation of ~3.

4. Lines 164-165: The statement "This distinction was visually corroborated by NMDS (Figure 2A) and PCoA (Figure 2B) plots" is not accurate at all- readers cannot even tell

which data points come from the same sample, so how can there be any judgement of stability? Please delete.

5. Figure 2: The large number of samples make it difficult to see the individual data points. Could the colours be made semi-transparent to show where multiple data points overlap? It also appears that there are only 8 samples in the 3rd trimester beta diversity plots. I assume this is also due to overlapping data points?

6. Line 166: “As defined in the methods,” is unnecessary.

7. Figure 3: I found it quite difficult to distinguish between the white page background and the low relative abundance values in the heatmap. I would ask the authors to consider changing the colour scale so that the colour scale goes from black, to yellow, to red, as abundance increases.

8. Lines 210-212: The finding regarding *Mycoplasma hominis* is something I don't disbelieve, as it has been reported by several other groups, but Table 4 and Figure 4 shows that the number of samples with detectable *M. hominis* is incredibly small and is in some cases 1 vs. 0. This limitation needs to be discussed. How do the 16S and qPCR data for *M. hominis* (and *U. urealyticum*) line up?

Reviewer #1 (Comments for the Author):

My detail comments for the present manuscript were listed as below:

(1) Although the current **Introduction** concludes by mentioning the aim to "move beyond the CST framework," it does not explicitly state testable research hypotheses (e.g., "We hypothesize that sPTB is associated with reduced temporal stability of the vaginal microbiome and enrichment of specific pathobionts, rather than CST type per se"). We recommend clearly listing 1-2 core hypotheses in the final paragraph to enhance the focus and clarity of the study design.

Response: Thank you for your kind suggest, we've revised the last paragraph of the introduction to make our study design more clear as below:

"To address this gap, we conducted a prospective longitudinal cohort study in a Chinese population. We employed a dual-method approach (16S rRNA sequencing and targeted qPCR) across three trimesters to characterize the vaginal microbiome's dynamic relationship with sPTB. Moving beyond the traditional CST framework, which often yields inconsistent associations, we focus on identifying some more specific microbial species and quantities, as well as temporal stability of the vaginal microbiome across pregnancy associated with sPTB."(Page 3, Lines 83-88)

(2) The authors are encouraged to cite prior studies on the vaginal microbiome in Asian or Chinese populations (if available) in the Introduction to justify why validating Western-derived microbial models in this specific cohort is particularly necessary. This would better highlight the novelty and gap-filling value of the present work.

Response: We thank the reviewer for this helpful suggestion. Following the reviewer's advice, we have revised the Introduction to include a discussion of prior studies in Chinese populations. While several studies in Chinese populations have indeed investigated the relationship between the vaginal microbiome and preterm birth, the findings have been inconsistent, and crucially, no study has yet examined the longitudinal dynamics of the vaginal microbiome across pregnancy in relation to sPTB in a Chinese cohort. This gap highlights the novelty and necessity of our study. The revised text now clearly states this rationale as below:

"In recent years, several studies have proved relationship between the vaginal microbiome and preterm birth in Chinese populations [17-20]. However, the findings have been notably inconsistent, particularly regarding alpha diversity and specific taxa associated with sPTB. More importantly, all existing studies in Chinese populations to date have employed cross-sectional or case-control designs with single time-point sampling. While these studies establish that associations exist, they cannot capture the dynamic nature of the vaginal microbiome during pregnancy or determine whether temporal instability, rather than a static community state, contributes to sPTB risk." (Page 3, Lines 75-82)

(3) In the differential abundance analyses, multiple statistical tests were performed across numerous taxa and time points, yet the manuscript does not mention whether corrections for multiple comparisons (e.g., FDR or Bonferroni) were applied. This omission may inflate the false discovery rate. Please clarify the correction strategy used, or explicitly label uncorrected P-values as such when interpreting results.

Response: We thank the reviewer for this critical methodological point. We have completely re-analyzed all differential abundance data using MaAsLin3, which implements Benjamini-Hochberg

(BH) false discovery rate (FDR) correction. This analysis was performed on both 16S rRNA sequencing data and targeted qPCR data (both abundance and prevalence). All reported associations now reflect q-values after FDR correction. The Methods section has been updated to describe this approach(Page6, Lines 152-171), and the Results now report corrected q-values throughout. Uncorrected P-values are clearly labeled as "nominally significant" to avoid confusion.

(4) CST classification itself remains a contentious approach in the field. We suggest that the authors complement their CST analysis with enterotype-like clustering (or alternative community stratification methods) tailored to their own dataset, and report the resulting distribution patterns to provide a more robust ecological characterization.

Response: We agree with the reviewer's concern. We have now re-performed CST classification using a data-driven enterotype-like clustering approach. Specifically, we used QIIME2 to generate species-level taxonomic profiles, calculated Bray-Curtis distances, and applied Ward.D2 hierarchical clustering to define CSTs within our own dataset. This approach ensures that the CST definitions are tailored to our study population rather than being forced into pre-defined categories from other cohorts(Page 5-6, Lines 146-151, Page 10-11, Lines 299-309). The Methods and Results have been updated using the customized CST.

(5) The Results section indicates significant differences between the sPTB and term groups in maternal age, BMI, and history of adverse obstetric outcomes. However, the primary analyses (e.g., CST stability and specific taxon abundance) appear not to have been adjusted for these potential confounders via multivariable regression. We strongly recommend incorporating covariate-adjusted models to strengthen causal inference.

Response: We thank the reviewer for this important observation. In our revised analysis using MaAsLin3, we have incorporated multivariable regression models that include maternal age, BMI, history of adverse obstetric outcomes, and parity as covariates. This adjustment strengthens the causal inference of our findings. The updated Methods and Results now reflect this covariate-adjusted approach. And the main change after multivariable regression was that no individual taxon remained significantly associated with sPTB and all nominally significant taxa had very low abundance (<1%) and failed to survive multiple testing correction.

(6) The quality and informativeness of the figures in this manuscript are suboptimal. We encourage the authors to include more customized or advanced visualizations (e.g., longitudinal trajectory plots, network analyses, or effect size displays) to extract and communicate deeper biological insights from their rich dataset.

Response: We thank the reviewer for this constructive suggestion. We have significantly enhanced our visualizations:

1.Compositional biplots have been added to PCoA plots (Figure 2) to show taxon loadings driving sample separation.

2.Sankey diagrams have been created to visualize CST transitions across trimesters for all participants (Figure 4), clearly demonstrating the reduced temporal stability in the sPTB group.

3.Stacked area graphs of community composition over three trimesters have been generated for all samples and all 9 sPTB cases with complete samples and 9 randomly selected term controls (Figure 5 and Figure 6), illustrating individual-level instability.

Reviewer #2 (Comments for the Author):

I enjoyed reading the study submitted by Yu and colleagues and applaud their collection of a valuable longitudinal dataset. I have the following comments which must be addressed:

Major issues

1. There is no explicit statement of: 1) ethical approval being granted for this study; 2) informed consent being sought from patients; and 3) the sequencing data being deposited in a publicly available repository. I am assuming that this was an accidental oversight by the authors, but all three MUST be provided. This is a requirement of Microbiology Spectrum.

Response: Thank you for your important suggestions. The description about the ethical approval and the informed consent has been added in the Study design and patient enrollment(Page 5, Lines 102-104)**.** The datasets used and analyzed during the current study are available from the China National Center for Bioinformation (CNCB), under GSA:CRA037472. You can access it through the following link: https://ngdc.cncb.ac.cn/gsa/s/265p0gtR.(Page 15, Lines 410-413)

2. The authors have chosen to take an OTU clustering approach (in QIIME1) rather than a more modern approach of using amplicon sequence variants (using QIIME2), which isn't inherently disqualifying, but their method for taxonomic assignment is contradictory. **Line 118** states that they used the GreenGenes database (which hasn't been updated since 2013) but **line 121** states that taxonmy was assigned via BLAST search against the "NCBI 16S rRNA" database. Which method was actually used, and with which version of the database? Was the NCBI database they refer to the one available at:https://ftp.ncbi.nlm.nih.gov/refseq/TargetedLoci/Bacteria/ ? These details need to be added to the methods. The authors should also consider the use of older approaches in their future work. Species level resolution with v3/v4 sequences is difficult, even more so when clustering at 97 % similarity (https://doi.org/10.1093/bioinformatics/bty113; essentially, the 97% threshold is outdated and too low for assigning species).

Response: We thank the reviewer for this important methodological critique. We have completely re-analyzed our 16S rRNA sequencing data using QIIME2. For taxonomic assignment, we used the NCBI 16S rRNA RefSeq database with a BLAST-based consensus approach. We have clarified this in the Methods (Page 5, Lines 142-145). And thanks for sharing this article. It clearly points out the importance of methodological development. In future research, we plan to adopt higher-resolution metagenomic sequencing technology. This not only can solve the problem of species-level identification, but also can deeply explore the functional potential of the microbiota, thereby more precisely clarifying the relationship between the vaginal microbiome and preterm birth.

3. The differential abundance analysis performed by the analysis is incredibly simplistic; a comparison of raw relative abundances (without adjusting for multiple comparisons!) is not appropriate when a multitude of dedicated tools for determining differential abundance exist. The authors must conduct a formal differential abundance analysis using purpose-built tools (e.g. ANCOM-BC, MaAsLin3, ALDEx2). I will leave the choice of tool up to the authors but reiterate that their current analysis is insufficient.

Response: We fully agree. We have now performed formal differential abundance analysis using MaAsLin3, a purpose-built tool for microbiome differential abundance testing. This analysis includes BH FDR correction for multiple comparisons and adjustment for potential confounders (maternal age, BMI, obstetric history, parity). The results are now reported with q-values, providing robust statistical inference.(Page 6, Lines 152-171)

4. The authors do not give any detail as to how the qPCR data in Table 3 has been processed. Table 3 shows the relative abundance of the species as determined by qPCR but there is no explanation in the methods section as to how this was calculated. Proportional abundance of the summed copy number per mL? This needs to be stated clearly in the methods and in the table legend. Moreover, the data in Tables 3 and 4 are not discussed chronologically. The authors go back and forth discussing data from each table. Please re-write **lines 177 - 185** so that all the observations from Table 3 are discussed before those from Table 4.

Response: We thank the reviewer for these clarifications. We have now added detailed qPCR processing information to the Methods: “For qPCR quantification, a plasmid containing the specific microbial targets and 16S PCR amplicon from E. coli was serially diluted from 107 to 10 copies to generate a standard curve and determine qPCR efficiency. The concentrations of microorganisms and 16S were derived from the measured Ct values, enabling subsequent calculation of their relative percentages in the samples.”(Page 5, Lines 126-130)

We have also reorganized the Results section to discuss abundance data (Table 3) first, followed by prevalence data (Table 4), ensuring chronological and logical flow. The table legends have been updated to clearly indicate that MaAsLin3 with FDR correction was used for statistical analysis.(Page 9, Lines 262-269)

5. The language used by the authors to describe some of their findings is far too strong and 'oversells' their data. For example, these P values in lines 157-158 regarding alpha diversity metrics in Figure 1 are nowhere close to a 'trend'. A far more accurate statement is, "there was no significant difference between term and sPTB groups in terms of alpha diversity by any metric tested", which is what their data shows. Likewise, ANOSIM may well be returning a significant P value, but the NMDS and PCoA plots show a complete overlap of the term and sPTB groups at every trimester. All of the plots in Figure 2A and 2B (why are these on separate pages?) show a clear separation of the samples into three main groups, which is not even discussed. What is the driver of this separation? Presumably it corresponds to lacto-dominant vs. lacto-deficient samples? I would like to see this discussed- perhaps labels showing the loadings of the taxa could be added (i.e. a compositional biplot).

Response: We appreciate this feedback and have tempered our language throughout the manuscript. We now state: "There were no significant differences between term and sPTB groups in any alpha diversity metric at any trimester after FDR correction (all $q > 0.05$)"(Page 7, Lines 196-198). Regarding beta diversity, we have added a more nuanced interpretation: "PCoA and NMDS visualization (**Figure 2**) showed substantial overlap between term and sPTB groups at all time points. Within-group comparisons revealed no significant differences in community composition across trimesters for either the term or sPTB group (all $q > 0.05$)." (Page 7, Lines 203-206). We have also added compositional information to explain the separation observed in PCoA plots. The three main

clusters are driven by dominance of Lactobacillus, Gardnerella, and Streptococcus, respectively. We have now investigated the drivers of this separation and incorporated this results and discussion into the revised manuscript.(Page 7-8, Lines 207-219, Page 11, Lines 310-323)

6. Similar to the point above, I do not think that the way that the authors have presented the beta-diversity data (as PCA/nMDS plots) shows 'instability' as one cannot see the samples from the same patient across time on the same plot. Instability would suggest that the community is constantly changing. Longitudinal data is very valuable, and the authors have gone to great lengths to collect these samples, yet they are not making the most of it at present. To make any comment about instability, the authors could (and should) create stacked area graphs of the community composition (e.g. relative abundance) over the three trimesters they have measured. This could be done in R quite easily using ggplot2, and a separate plot produced for each of the 26 sPTB samples (e.g. using ggplot's faceting feature). See https://r-graph-gallery.com/136-stacked-area-chart.html for an example. A similar figure could be produced for 26 random term samples for comparison.
Response: Excellent suggestion. We have generated stacked area graphs of community composition over three trimesters for all cases and 9 sPTB cases with complete longitudinal samples and 9 randomly selected term controls. These are now presented as Figure5 and Figure 6. The stacked area plots clearly illustrate marked compositional fluctuations in sPTB cases compared to relatively stable communities in term controls. For example, several sPTB cases show dramatic shifts from Lactobacillus dominance to mixed communities and back, while term controls typically maintain stable dominance of a single Lactobacillus species throughout pregnancy. This visualization provides direct evidence for the instability we describe and has been added to the Results and Discussion(Page 8-9, Lines 240-251, Page 11, Lines 299-309).

7. Where the authors do explicitly mention stability, the data presented is limited. This data is important and should be presented in the paper. I ask that the authors create two sankey diagrams (or some other visualisation) showing the change in CST assignments of each individual across the three trimesters, one for the term group and another for the sPTB group. See https://r-graph-gallery.com/sankey-diagram.html for examples.
Response: Based on Reviewer1' suggestion, we have now re-performed CST classification using a data-driven enterotype-like clustering approach. Specifically, we used QIIME2 to generate species-level taxonomic profiles, calculated Bray-Curtis distances, and applied Ward.D2 hierarchical clustering to define CSTs within our own dataset, as shown in **Figure 3**. This approach ensures that the CST definitions are tailored to our study population rather than being forced into pre-defined categories from other cohorts(Page 5-6, Lines 146-151, Page 8, Lines 222-229). According to the new CST clustering, we have created Sankey diagrams illustrating CST transitions across trimesters for all participants, with separate diagrams for term and sPTB groups. These are now presented as **Figure 4**. The visualizations clearly demonstrate that 182 of women in the term group maintained the same CST throughout pregnancy, compared to only 5 in the sPTB group ($P < 0.05$, Fisher's exact test). This compelling visualization now appears in the Results and provides support for our hypothesis regarding temporal instability. The Sankey diagrams also reveal patterns of transition

between specific CSTs, showing that sPTB cases more frequently transition from Lactobacillus-dominated states to mixed communities and back.(Page 8, Lines 235-239)

Minor issues

1. Line 57: was intra-amniotic infection documented in any of the 26 PTB cases? If so, was any culture attempted? Causative organisms? This would be interesting to know and to see if the 16S or qPCR data corroborate this.

Response: We appreciate the reviewer's interest in the potential role of intra-amniotic infection in our sPTB cases. Amniotic fluid samples were collected during delivery from a subset of the preterm birth cases. However, due to the clinical circumstances of preterm delivery (particularly in cases with PPROM or rapid progression of labor), obtaining amniotic fluid samples was not feasible for all 26 sPTB cases. For those cases where amniotic fluid was successfully collected, we performed conventional bacterial culture using standard culture media. No pathogenic bacteria were cultured from any of the amniotic fluid samples tested.

2. Line 112: which kit was used for library preparation?

Response: We thank the reviewer for the thoughtful suggestion.we have added details in the Method "DNA templates were amplified using 1× KAPA HiFi Master Mix (Cat. No. 16SAFP02; Coyote Biosciences, Beijing, China), 0.01 nmol of each primer, and 9 μL genomic DNA. Amplification products were purified with VAHTS clean beads (Na44-02; Coyote) and quantified with a Qubit dsDNA HS Assay Kit (Q32851; Life Technologies). Amplicon libraries were prepared, purified, and paired-end sequenced (2×250 bp) on an Illumina HiSeq 2500 platform (Illumina Inc, San Diego, CA)."(Page 5, Lines 134-139)

3. Lines 148-149: Have the 17 P values in Table 1 been adjusted for multiple testing? Many differences between groups are relatively small with a large standard deviation e.g. BMI of 22.1 vs 21.4 both with a standard deviation of ~3.

Response: We thank the reviewer for raising this important point. The P-values presented in Table 1 are uncorrected for multiple testing, as is standard practice for demographic and clinical characteristics tables in clinical research. These variables are not considered primary outcomes but rather descriptive characteristics of the study population, and the P-values are intended to describe the cohort composition rather than to test formal hypotheses. The reviewer correctly notes that some differences, such as BMI (22.1 ± 3.3 vs. 21.4 ± 3.1, $P < 0.001$), show statistically significant P-values despite relatively small absolute differences. This reflects the large sample size in the term group (n=247), which provides high statistical power to detect even modest differences. The clinical significance of these small absolute differences is debatable, but their statistical significance in univariate comparisons justifies their inclusion as covariates in multivariable models. However, we acknowledge the reviewer's concern about the risk of Type I error when interpreting multiple comparisons. We have now added a footnote to Table 1 clarifying that these are uncorrected P-values and have revised the text to focus on the role of these variables as confounders in multivariable models rather than as independent findings.

4. Lines 164-165: The statement "This distinction was visually corroborated by NMDS (Figure 2A)

and PCoA (Figure 2B) plots" is not accurate at all- readers cannot even tell which data points come from the same sample, so how can there be any judgement of stability? Please delete.
Response: We thank the reviewer for this helpful suggestion, and we've deleted it.

5. Figure 2: The large number of samples make it difficult to see the individual data points. Could the colours be made semi-transparent to show where multiple data points overlap? It also appears that there are only 8 samples in the 3rd trimester beta diversity plots. I assume this is also due to overlapping data points?
Response: We thank the reviewer for this helpful suggestion to improve the clarity of Figure 2. We have adjusted all data points in the PCoA plots to 50% transparency (alpha = 0.5) and reordered the plotting sequence so that sPTB samples (red) are plotted on top of term samples (blue) , ensuring that preterm cases remain visible even when they co-occur with a large number of term samples.
The reviewer correctly observes that only approximately 8 sPTB samples appear visible in the third trimester PCoA plot. This is due to two factors: First, the sPTB group sample size in the third trimester is n=10 (not 8). The reduction in sample size in the sPTB group during the third trimester is an inherent and unavoidable consequence of the study design, given that spontaneous preterm birth, by definition, occurs before 37 weeks of gestation. Women who delivered preterm in the second or early third trimester could not contribute samples at later time points. This limitation reflects the biological reality of the condition rather than a methodological flaw. We have acknowledged this limitation in the Discussion and have taken care to interpret third-trimester findings with appropriate caution given the reduced sample size(Page 12-13, Lines 360-370). Second, with the semi-transparent rendering now applied, the density of sPTB samples in this region becomes more apparent, as the red coloration darkens with increasing overlap.

6. Line 166: "As defined in the methods," is unnecessary.
Response: We appreciate the reviewer's suggestion, and we've revised it.

7. Figure 3: I found it quite difficult to distinguish between the white page background and the low relative abundance values in the heatmap. I would ask the authors to consider changing the colour scale so that the colour scale goes from black, to yellow, to red, as abundance increases.
Response: We thank the reviewer for this helpful suggestion to improve the readability of Figure3. The color has been reset according to your suggestion.

8. Lines 210-212: The finding regarding Mycoplasma hominis is something I don't disbelieve, as it has been reported by several other groups, but Table 4 and Figure 4 shows that the number of samples with detectable M. hominis is incredibly small and is in some cases 1 vs. 0. This limitation needs to be discussed. How do the 16S and qPCR data for M. hominis (and U. urealyticum) line up?
Response: We thank the reviewer for this important observation. We have now:
Explicitly discussed this limitation in the Results: "For example, *M. hominis* was detected in only 1/225 term and 1/20 sPTB samples in the second trimester, and *B. fragilis* in 1/225 term and 1/20 sPTB samples, making robust statistical inference impossible."(Page 9-10, Lines 269-275)
Added to the Discussion: "The discrepancy between 16S and qPCR detection of low-abundance organisms like *M. hominis* highlights the complementary value of targeted molecular approaches. 16S

sequencing failed to detect *M. hominis* in any sample, likely due to its low abundance falling below the sequencing depth detection threshold, while qPCR successfully identified it in a small number of samples. This suggests that targeted approaches may be necessary to detect rare but potentially pathogenic organisms, and that 16S-based studies may underestimate the role of low-abundance taxa. However, the extremely low detection frequency also means that even qPCR may lack sensitivity for population-level studies unless sample sizes are very large. Future studies should consider enrichment strategies or deeper sequencing to capture rare taxa."(Page 12, Lines 343-352)

Re: Spectrum03705-25R1 (**Longitudinal Changes in the Vaginal Microbiome Associate with Spontaneous Preterm Birth: A Focus on Stability and Specific Taxa in a Chinese Cohort**)

Dear Dr. Juntao Liu:

Thank you for the privilege of reviewing your work. Below you will find my comments, instructions from the Spectrum editorial office, and the reviewer comments.

Please address the concerns raised by Reviewer 2.

Please return the manuscript within 60 days; if you cannot complete the modification within this time period, please contact me. If you do not wish to modify the manuscript and prefer to submit it to another journal, notify me immediately so that the manuscript may be formally withdrawn from consideration by Spectrum.

Revision Guidelines
To submit your modified manuscript, log into the submission site at https://spectrum.msubmit.net/cgi-bin/main.plex. Go to Author Tasks and click the appropriate manuscript title to begin. The information you entered when you first submitted the paper will be displayed; update this as necessary. Note the following requirements:

• Upload point-by-point responses to the issues raised by the reviewers in a file named "Response to Reviewers," NOT in your cover letter.
• Upload a compare copy of the manuscript (without figures) as a "Marked-Up Manuscript" file.
• Upload a clean .DOC/.DOCX version of the revised manuscript and remove the previous version.
• Each figure must be uploaded as a separate, editable, high-resolution file (TIFF or EPS preferred), and any multipanel figures must be assembled into one file.
• Any supplemental material intended for posting by ASM should be uploaded with their legends separate from the main manuscript. You can combine all supplemental material into one file (preferred) or split it into a maximum of 10 files with all associated legends included.

For complete guidelines on revision requirements, see our Submission and Review Process webpage. Submission of a paper that does not conform to guidelines may delay acceptance of your manuscript.

Data availability: ASM policy requires that data be available to the public upon online posting of the article, so please verify all links to sequence records, if present, and make sure that each number retrieves the full record of the data. If a new accession number is not linked or a link is broken, provide Spectrum production staff with the correct URL for the record. If the accession numbers for new data are not publicly accessible before the expected online posting of the article, publication may be delayed; please contact production staff (Spectrum@asmusa.org) immediately with the expected release date.

Publication Fees: For information on publication fees and which article types are subject to charges, visit our website. If your manuscript is accepted for publication and any fees apply, you will be contacted separately about payment during the production process; please follow the instructions in that e-mail. Arrangements for payment must be made before your article is published.

ASM Membership: Corresponding authors may join or renew ASM membership to obtain discounts on publication fees. Need to upgrade your membership level? Please contact Customer Service at Service@asmusa.org.

The ASM Journals program strives for constant improvement in our submission and publication process. Please tell us how we can improve your experience by taking this quick Author Survey.

Thank you for submitting your paper to Spectrum.

Sincerely,
Zhe Li
Editor
Microbiology Spectrum

Reviewer #1 (Comments for the Author):

Thanks for addressing my comments

Reviewer #2 (Comments for the Author):

Having read the authors revised manuscript, I have the following comments:

1. The loadings for the PCoA/nMDS plots are described in a way that is not consistent with what is shown in Figure2. The authors talk about cluster 1 being located on the left-hand side of the plots "in PCoA space", but this is only true for the nMDS plots, not the PCoA plots. Moreover, they describe cluster 2 as being located on the upper right-hand side of the plots and characterised by an abundance of Gardnerella; however, the cluster with an abundance of Gardnerella is on the upper-left for the PCoA plots at trim 2/3 and the lower-left for trimester 1. In the nMDS plots, it is located at the lower right-hand side of the plot. This needs to be re-written to reflect the actual figures themselves.

2. I realise the authors have re-done their CST analysis due to a comment by reviewer #1 about CSTs being a contentious approach in the field, but the heatmap from their clustering analysis in figure 3 now shows that the single largest cluster in their data (cluster I) is dominated by "atypical Lactobacillus subtypes" (line 222). This is incredibly different from the data in their initial submission showing that CST I (L. crispatus) accounted for most of the samples, and now L. crispatus is not even shown on the heatmap. This is despite lines 210-211 mentioning a particularly high relative abundance of L. crispatus and lines 241-243 mentioning that "Term controls typically maintain stable dominance of a single Lactobacillus species (predominantly L. crispatus or L. iners) throughout pregnancy". Why the discrepancy? In their response to my initial comment #7, the authors say that they "used QIIME2 to generate species-level taxonomic profiles", yet they are showing a genus-level taxon in figure 3. Is their cluster I not still L. crispatus?

3. Can the stacked area graphs split Lactobacillus into the distinct species commonly seen in the vaginal environment? Presently, aggregating all lactobacilli at the genus level obfuscates species-level changes in the lactobacilli that the Sankey diagrams show.

4. The differential abundance analysis using MaAsLin3 now presented by the authors is explained in a contradictory manner to what figure 7 shows. Fig 7 shows that the only taxa significantly associated with pre-term birth is Bifidobacterium breve (indicated by double asterisks, which in maaslin3 represents a q value lower than the q-value significance threshold divided by 10). This seems to be reflected in the data from figure 6, showing two individuals (S032 and S129) in the sPTB group whose vaginal microbiomes contain lots of Bifidobacterium (presumably most of which is B. breve). This is the opposite of what is currently described on lines 256-260: "Except for Bifidobacterium breve taxa showed significant associations in the second trimester between two groups after FDR correction, all the others had q-values > 0.1 after multiple testing correction". Is there a typo here? Should this not be "Except for B. breve, taxa showed no significant associations....". Equally, I am not quite sure why the authors describe this heatmap of the coefficients as a forest plot? In any case, the authors must clarify their intended meaning. It would also be helpful to add a sentence to the legend of figure 7 stating in which direction the difference lies (i.e. B. breve is overrepresented in sPTB group).

Reviewer #2 (Comments for the Author):

Having read the authors revised manuscript, I have the following comments:

1.The loadings for the PCoA/nMDS plots are described in a way that is not consistent with what is shown in Figure2. The authors talk about cluster 1 being located on the left-hand side of the plots "in PCoA space", but this is only true for the nMDS plots, not the PCoA plots. Moreover, they describe cluster 2 as being located on the upper right-hand side of the plots and characterised by an abundance of Gardnerella; however, the cluster with an abundance of Gardnerella is on the upper-left for the PCoA plots at trim 2/3 and the lower-left for trimester 1. In the nMDS plots, it is located at the lower right-hand side of the plot. This needs to be re-written to reflect the actual figures themselves.

Response: We thank the reviewer for this careful observation. We acknowledge that our original description of cluster locations relative to the PCoA and NMDS axes was inaccurate and did not consistently reflect the actual figures. To address this, we have removed all subjective spatial descriptions (e.g., "left side", "upper right") from the Results and Figure 2 legend. Instead, we now describe the clusters solely based on their driving taxa as determined by species loading analysis, without reference to their position in the ordination space. Specifically, we have revised the text to state:"Cluster 1 was characterized by high relative abundance of *Lactobacillus* species, particularly *L. crispatus* and *L. iners*, and corresponded to *Lactobacillus*-dominant CSTs. Cluster 2 was driven by high abundance of *Gardnerella vaginalis*, often accompanied by *Atopobium vaginae, Prevotella spp.*, and other BV-associated taxa, corresponding to high-diversity CSTs. Cluster 3 was associated with *Streptococcus spp*. and other opportunistic pathogens (e.g., *Ureaplasma, Staphylococcus, Enterococcus*), representing a more heterogeneous group of non-canonical vaginal communities. " (Lines 211-218). The corresponding legend in Figure 2 has also been revised accordingly. We believe this revision eliminates the inconsistency and focuses the reader's attention on the biological drivers of the separation rather than their spatial orientation, which can vary depending on the ordination method and axes.

2. I realise the authors have re-done their CST analysis due to a comment by reviewer #1 about CSTs being a contentious approach in the field, but the heatmap from their clustering analysis in figure 3 now shows that the single largest cluster in their data (cluster I) is dominated by "atypical Lactobacillus subtypes" (line 222). This is incredibly different from the data in their initial submission showing that CST I (L. crispatus) accounted for most of the samples, and now L. crispatus is not even shown on the heatmap. This is despite lines 210-211 mentioning a particularly high relative abundance of L. crispatus and lines 241-243 mentioning that "Term controls typically maintain stable dominance of a single Lactobacillus species (predominantly L. crispatus or L. iners) throughout pregnancy". Why the discrepancy? In their response to my initial comment #7, the authors say that they "used QIIME2 to generate species-level taxonomic profiles", yet they are showing a genus-level taxon in figure 3. Is their cluster I not still L. crispatus?

Response: We thank the reviewer for identifying this critical discrepancy. We have carefully re-examined our analytical pipeline and would like to provide a transparent explanation of the differences between our initial and revised analyses.

In our initial data analysis, we used QIIME1 based on raw 16S rRNA gene sequences. High-quality sequences were obtained after quality filtering, merging of paired-end reads, and removal of chimeras. Operational Taxonomic Units (OTUs) were clustered at 97% similarity, and taxonomic information was annotated against the NCBI 16S rRNA database. This approach allowed us to perform subspecies-level analysis on the Lactobacillus genus sequences by cross-referencing with published literature. Using this method, L. crispatus was successfully identified as the dominant species in the largest cluster. In response to reviewer comments regarding methodological rigor, we completely re-analyzed all 16S data using QIIME2 (version 2022.8) . Raw sequences were processed using the DADA2 pipeline within QIIME2, which generates amplicon sequence variants (ASVs) rather than traditional OTUs. This approach resolves sequences at single-nucleotide resolution, providing substantially higher resolution than the 97% OTU clustering method. ASVs were taxonomically classified using the SILVA 138 reference database, since SILVA is currently one of the most comprehensive public databases for the 16S rRNA gene, featuring the advantages of timely updates and comprehensive coverage. Critically, using this new analytical pipeline (QIIME2 + DADA2 + SILVA 138), L. crispatus was not annotated in the results and therefore does not appear in the revised heatmap (Figure 3). We have investigated this issue and identified the most likely explanation: the SILVA database exhibits certain insufficient coverage of the species sequences of Lactobacillus in the reproductive tract, leading to the presence of certain Lactobacillus taxa that cannot be clearly identified. To address this, we validated species assignments for the Lactobacillus using BLAST against the NCBI 16S rRNA database, confirming that the largest CST cluster was dominated by L. crispatus. We've rewrite the manuscript as below: " The Lactobacillus species was cross-validated via BLAST search against the NCBI 16S rRNA database (https://ftp.ncbi.nlm.nih.gov/blast/db/16S_ribosomal_RNA.tar.gz)."(Lines 145-147). "Our data-driven approach identified six CSTs with the following dominant taxa: CST I (Dominated by *Lactobacillus crispatus*)"(Lines 222-223). And cluster I is still L. crispatus in Figure3.

Figure 3 displays the classification results at the L7 level, which represents the most detailed taxonomic assignment achievable for each ASV based on the SILVA 138 database. The L7 level corresponds to the lowest taxonomic rank that can be reliably assigned, typically species level when sufficient resolution is available, but may fall back to genus level or higher when species-level assignment is ambiguous. Our statement that we "used QIIME2 to generate species-level taxonomic profiles" refers to our analytical intent and the capability of the pipeline to assign species-level taxonomy when sequence resolution permits. However, we acknowledge that not all ASVs can be confidently assigned to the species level, which is why we present the L7 level results as generated by the classifier rather than forcing all assignments to species level.

3.Can the stacked area graphs split Lactobacillus into the distinct species commonly seen in the vaginal environment? Presently, aggregating all lactobacilli at the genus level obfuscates species-level changes in the lactobacilli that the Sankey diagrams show.

Response: Thank you for your constructive suggestion. We've redrawn Figure 5 and Figure6 , dividing Lactobacillus into five types for drawing: Lactobacillus crispatus, Lactopacillus gassen, Lactobacillus iners, Lactobacillus jensen, and Lactobacillus delbrueckii.

4.The differential abundance analysis using MaAsLin3 now presented by the authors is explained in a contradictory manner to what figure 7 shows. Fig 7 shows that the only taxa significantly associated with pre-term birth is **Bifidobacterium breve** (indicated by double asterisks, which in maaslin3 represents a q value lower than the q-value significance threshold divided by 10). This seems to be reflected in the data from figure 6, showing two individuals (S032 and S129) in the sPTB group whose vaginal microbiomes contain lots of Bifidobacterium (presumably most of which is **B. breve**). This is the opposite of what is currently described on lines 256-260: "Except for Bifidobacterium breve taxa showed significant associations in the second trimester between two groups after FDR correction, all the others had q-values > 0.1 after multiple testing correction". Is there a typo here? Should this not be "Except for B. breve, taxa showed no significant associations....". Equally, I am not quite sure why the authors describe this heatmap of the coefficients as a forest plot? In any case, the authors must clarify their intended meaning. It would also be helpful to add a sentence to the legend of figure 7 stating in which direction the difference lies (i.e. B. breve is overrepresented in sPTB group).

Response: We thank the reviewer for catching this critical error in our description. We've rewrite lines 256-260 as below: "Except for *Bifidobacterium breve*, taxa showed no significant associations in the second trimester between two groups after FDR correction, all the others had q-values > 0.1 after multiple testing correction. Importantly, the significant taxa had very low prevalence and abundance (< 1% relative abundance in most cases), limiting its biological and clinical significance.". And the legend of Figure7 has been rewrite as below: "**Figure 7. Differential abundance analysis.** Heatmap showing taxa with nominally significant associations with spontaneous preterm birth (sPTB) in the second trimester (22-28 weeks) from MaAsLin3 analysis. The color scale indicates prevalence and abundance, with both set to 1.0 for all displayed taxa. *Bifidobacterium breve* is significantly overrepresented in sPTB group，while no other taxa showed significant associations in the second trimester between two groups after FDR correction, all the others had q-values > 0.1 after multiple testing correction."(lines 612-618)

Re: Spectrum03705-25R2 (**Longitudinal Changes in the Vaginal Microbiome Associate with Spontaneous Preterm Birth: A Focus on Stability and Specific Taxa in a Chinese Cohort**)

Dear Dr. Juntao Liu:

Your manuscript has been accepted, and I am forwarding it to the ASM production staff for publication. Your paper will first be checked to make sure all elements meet the technical requirements. ASM staff will contact you if anything needs to be revised before copyediting and production can begin. Otherwise, you will be notified when your proofs are ready to be viewed.

Data Availability: ASM policy requires that data be available to the public upon online posting of the article, so please verify all links to sequence records, if present, and make sure that each number retrieves the full record of the data. If a new accession number is not linked or a link is broken, provide production staff with the correct URL for the record. If the accession numbers for new data are not publicly accessible before the expected online posting of the article, publication may be delayed; please contact ASM production staff immediately with the expected release date.

Publication Fees: For information on publication fees and which article types have charges, please visit ourwebsite. We have partnered with Copyright Clearance Center (CCC) to collect author charges. If fees apply to your paper, you will receive a message from no-reply@copyright.com with further instructions. For questions related to paying charges through RightsLink, please contact CCC at ASM_Support@copyright.com or toll free at +1-877-622-5543. CCC makes every attempt to respond to all emails within 24 hours.

ASM Membership: Corresponding authors may join or renew ASM membership to obtain discounts on publication fees. Need to upgrade your membership level? Please contact Customer Service at Service@asmusa.org.

Embargo Policy: A press release may be issued as soon as the manuscript is posted on theSpectrum Latest Articles webpage. The corresponding author will receive an email with the subject line "ASM Journals Author Services Notification" when the article is available online.

The ASM Journals program strives for constant improvement in our submission and publication process. Please tell us how we can improve your experience by taking this quick Author Survey.

Thank you for submitting your paper to Spectrum.

Sincerely,
Zhe Li
Editor
Microbiology Spectrum

Reviewer #2 (Comments for the Author):

Thanks very much for answering my questions & providing clarification.
